# Twenty-four new species of *Polycentropus* (Trichoptera, Polycentropodidae) from Brazil

**DOI:** 10.3897/zookeys.76.790

**Published:** 2011-01-19

**Authors:** Steven W. Hamilton, Ralph W. Holzenthal

**Affiliations:** 1 Austin Peay State University, The Center for Field Biology, P. O. Box 4718, Clarksville, Tennessee 37044, U.S.A.; 2 University of Minnesota, Department of Entomology, 1980 Folwell Ave., 219 Hodson Hall, St. Paul, Minnesota 55108, U.S.A

**Keywords:** Trichoptera, Polycentropodidae, *Polycentropus*, caddisfly, new species, Neotropics, Brazil

## Abstract

Twenty-four new species of the caddisfly genus Polycentropus (Insecta: Trichoptera: Polycentropodidae) occurring in Brazil are diagnosed, described, and the male genitalia of each are illustrated. Eighteen of the new species are placed in the Polycentropus jorgenseni species complex of the Polycentropus gertschi group of New World Polycentropus *sensu lato*. Furthermore, 6 new species within the Polycentropus gertschi group **(**Polycentropus ancistrus **sp. n.**, Polycentropus boraceia **sp. n.**, Polycentropus carioca **sp. n.**, Polycentropus froehlichi **sp. n.**, Polycentropus galharada **sp. n.**, and Polycentropus graciosa **sp. n.**) are placed in an informal diagnostic cluster of species with Polycentropus urubici Holzenthal and Almeida. Ten of the other Polycentropus gertschi group species form a second cluster of diagnostically similar species, the Polycentropus soniae cluster (Polycentropus caaete **sp. n.**, Polycentropus carolae **sp. n.**, Polycentropus cheliceratus **sp. n.**, Polycentropus fluminensis **sp. n.**, Polycentropus itatiaia **sp. n.**, Polycentropus minero **sp. n.**, Polycentropus santateresae **sp. n.**, Polycentropus soniae **sp. n.**, Polycentropus tripui **sp. n.**, and Polycentropus virginiae **sp. n.**). Two of the remaining 8 new species are included in the Polycentropus jorgenseni species complex (Polycentropus cipoensis **sp. n.** and Polycentropus verruculus **sp. n.**), while the remaining 6 are unique and cannot be placed in one of the groups at this time (Polycentropus acinaciformis **sp. n.**, Polycentropus amphirhamphus **sp. n.**, Polycentropus cachoeira **sp. n.**, Polycentropus inusitatus **sp. n.**, Polycentropus paprockii **sp. n.** and Polycentropus rosalysae **sp. n.**).

## Introduction

Although cosmopolitan, Polycentropus is still not well known is South America. Flint et al. (1999) reported 64 species of Polycentropus for the Neotropics. Subsequently, only 14 species in the genus have been added, 1 each from Argentina (Angrisano and Sganga 2009), Brazil (Holzenthal and Almeida 2003) and Hispaniola (Flint and Sykora 2004), 2 each from Costa Rica (Chamorro-Lacayo 2003), Ecuador, and Venezuela (Hamilton and Holzenthal 2005), and 5 from Mexico (Barba-Alvarez and Bueno-Soria 2005). In their checklist of the caddisflies of Brazil, Paprocki et al. (2004) reported 378 species as of September, 2003. More than half (26) of the 46 species of polycentropodids included, were Cernotina. To date, the only named species of Polycentropus from Brazil is Polycentropus urubici Holzenthal & Almeida (2003). No Polycentropus species have been added to the known Brazilian fauna since.

In this paper we add 24 new species to the list of Brazilian Polycentropus, 9 of which were previously described and illustrated in a dissertation on the New World species of Polycentropus *sensu stricto* (Hamilton 1986). The remaining 15 were collected as part of asurvey of the caddisflies of southeastern and southern Brazil by Dr. Ralph Holzenthal and colleagues.

## Materials and methods

All material described here is based on pinned specimens, except as noted. While preparation of male genitalia occurred over many years, the procedures for clearing and illustrating the specimens generally followed those presented by Holzenthal and Andersen (2004).

Terminology used in describing male genitalia follows that of Hamilton (1986) and, particularly in regard to naming the parts of the preanal appendage, Chamorro and Holzenthal (2010). Paired appendages and the processes of these appendages (intermediate appendages, preanal appendages, and inferior appendages) are referred to in the singular in diagnoses and descriptions. Our interpretation of the shape and position of the mostly membranous tergum IX + X should not be considered absolute because the membranous nature of the segments makes their demarcation difficult to discern and their exact shapes subject to degree of inflation at the time of preservation or during the clearing process. Likewise, the membranous attachment between the intermediate and preanal appendages as well as the shape of the endothecal membranes are variable and our descriptions represent our best interpretation of their relative positions and shapes in the specimens we have studied. Because of their small size, the shapes of the endothecal and subphallic sclerites are difficult to discern, especially if deeply embedded in membranes; in particular the shape of the pedicel of the subphallic sclerite, if present, and the length of the arms that cradle the phallobase are often hard to discern. In Polycentropus carioca sp. n., Polycentropus boraceia sp. n., Polycentropus galharada sp. n., Polycentropus graciosa sp. n., Polycentropus froehlichi sp. n., and Polycentropus ancistrus sp. n., the base of the mesolateral process of the preanal appendage is lightly sclerotized, so its position relative to the mesoventral process is not fixed.

Females that were collected at the same location and date as described males are included in the type series, although the certainty of these associations awaits further investigation. Types are deposited in the collections of the Museo de Zoologia, Universidade do São Paulo, São Paulo, Brazil (MZUSP), United States National Museum of Natural History, Washington, DC (NMNH), the Universidade Federal do Rio de Janeiro, Rio de Janeiro, Brazil (UFRJ), the Universidade Federal do Bahia, Salvador, Brazil (UFBA), and the University of Minnesota Insect Collection, St. Paul, Minnesota, U.S.A. (UMSP), as indicated in the species description. The holotypes of Polycentropus minero sp. n. and Polycentropus carolae sp. n., are on long term loan to the NMHN from MZUSP.

## Phylogenetic considerations

Eighteen of the 24 new Brazilian species are clearly members of the Polycentropus gertschi group (hereafter referred to as the *gertschi* group). This group, as characterized by Hamilton (1986), comprises most of the Neotropical Polycentropus species. The *gertschi* group shares 3 synapomorphies: “(1) ventral process of phallobase is apicoventral in position, (2) each intermediate appendage free to its base and articulates basomesally on its preanal appendage, and (3) there is a sclerite formed in the membrane below and around the phallus that appears to support and guide this organ” (Hamilton and Holzenthal 2005). These 18 species are further placed in the Polycentropus jorgenseni species complex (hereafter referred to as the *jorgenseni* species complex) within the *gertschi* group, a clade that Hamilton (1986) characterized based on the occurrence of a dorsal sclerotized band in the endothecal membranes (often folded back into phallobase when endothecal membrane inverted). The remaining 6 new species, including Hamilton’s (1986) “Polycentropus sp. n. 8” and “Polycentropus sp. n. 9,” are more problematic in regard to group affinities based on characters employed by Hamilton (1986). All 6 species appear to lack the articulated intermediate appendages so evident in members of the *gertschi* group, but some of the 6 have other characters that suggest affinities to the group, including the endothecal sclerotic band in Polycentropus rosalysae sp. n. Polycentropus acinaciformis sp. n., and Polycentropus cachoeira sp. n. In lieu of a phylogenetic analysis, discussion of suggested affinities will be included in the diagnoses of the species.

In the following descriptions, the first 6 species (Polycentropus ancistrus sp. n., Polycentropus boraceia sp. n., Polycentropus carioca sp. n., Polycentropus froehlichi sp. n., Polycentropus galharada sp. n., and Polycentropus graciosa sp. n.) seem to demonstrate affinities to Polycentropus urubici Holzenthal & Almeida (2003) based in particular on the shape of the preanal appendage. The mesolateral process of the preanal appendage is digitate and its base is narrowed and lightly sclerotized. As a result, its position relative to the rigid mesoventral process is not fixed. In addition, in these 6 species and Polycentropus urubici, the posteroventral region of the inferior appendage is short while the dorsolateral flange is relatively high and more elongate, forming a pointed process in most species. Also, these species have a vestiture of fine, black setae on the wings and lack the patches of pale setae so common in other Polycentropus. Unlike Polycentropus urubici, the intermediate appendage is long, often more than twice the length of the digitate mesolateral process of the preanal appendage. In the diagnoses below, we refer to this diagnostic cluster of 7 species as the Polycentropus urubici cluster (hereafter referred to as the *urubici* cluster), although we do not intend to indicate that a phylogenetic analysis has occurred nor that this cluster be recognized as a formal clade.

The next 10 species descriptions, also species of the *jorgenseni* species complex are close to the recently described Polycentropus aguyje Angrisano and Sganga (2009) (Polycentropus caaete sp. n., Polycentropus carolae sp. n., Polycentropus cheliceratus sp. n., Polycentropus fluminensis sp. n., Polycentropus itatiaia sp. n., Polycentropus minero sp. n., Polycentropus santateresae sp. n., Polycentropus soniae sp. n., Polycentropus tripui sp. n., and Polycentropus virginiae sp. n.). These appear similar in the compact, often quadrate, lateral aspect of the interior appendage; the elongate, typically decurved intermediate appendage; and the shape of the preanal appendage in lateral view with its mesolateral process short and very broad and the mesoventral process more digitate. As in the *urubici* cluster, the wings appear solid black and lack pale patches of setae. For convenience, we will designate this diagnostically similar cluster of 11 species as the Polycentropus aguyje cluster (hereafter referred to as the *aguyje* cluster). As in the *urubici* cluster discussed above, the *aguyje* cluster should not be recognized as a formal clade.

Of the remaining 8 new Brazilian species, 2 species are clearly members of the *jorgenseni* species complex (Polycentropus cipoensis sp. n., and Polycentropus verruculus sp. n.), but are unique and not similar to members of the *urubici* and *aguyje* clusters. The final 6 species (Polycentropus acinaciformis sp. n., Polycentropus amphirhamphus sp. n., Polycentropus cachoeira sp. n., Polycentropus inusitatus sp. n., Polycentropus paprockii sp. n., Polycentropus rosalysae sp. n.) cannot be placed in the *jorgenseni* species complex based on the synapomorphies identified by Hamilton (1986), although several show some similarities to the complex.

Ongoing investigations by Brazilian researchers and others (e.g., Chamorro and Holzenthal 2010) may add to our understanding of the phylogenetic relationships of these and other species of Neotropical Polycentropus. Clearly, Brazil has a poorly understood richness of species in the genus.

## Species descriptions

### Polycentropus boraceia

Hamilton & Holzenthal sp. n.

urn:lsid:zoobank.org:act:8B813827-03F9-4752-8D82-E0E79968F5F8

Fig. 1

Polycentropus  new species 7 Hamilton 1986: 93–94, 210; Fig. 6.10.

#### Description.

Similar to Polycentropus urubici Holzenthal & Almeida (2003) in general form, Polycentropus boraceia sp. n. is particularly distinct in the much greater length and shape of the intermediate appendage and the general shape of the inferior appendage. Most similar to Polycentropus galharada sp. n., the shape of the inferior appendage in lateral and posterior views provides distinctive features that separate Polycentropus boraceia sp. n. from Polycentropus galharada sp. n. as well as other species of the *urubici* cluster. In particular, the dorsolateral flange of the inferior appendage is shorter and more dorsally rounded in lateral view and the mesoventral spine in posterior aspect is acute while in Polycentropus galharada sp. n. this spine is more angularly truncate.

##### Adult.

Length of forewing (male) 6.1–8.8 mm. Body dark brown to black; dorsum of head and thorax dark brown, clothed with long, erect dark setae; base of forewing with long, erect dark setae, general vestiture of forewing with fine black setae, lacking patches of pale setae; legs dark brown to black.

##### Male.

**Figure 1.**
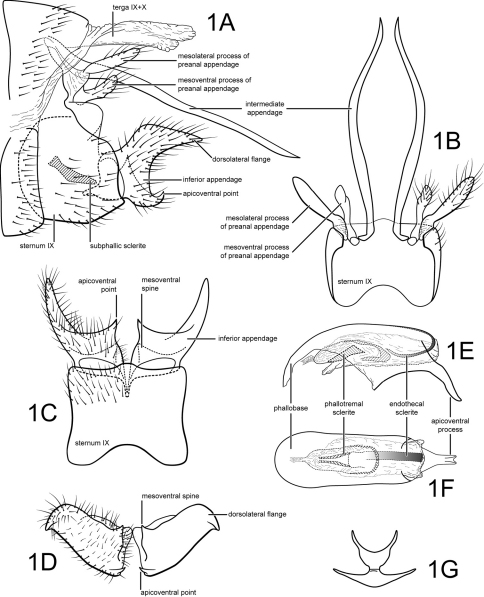
Polycentropus boraceia sp. n. Male genitalia: **A** lateral **B** dorsal **C** ventral **D** inferior appendages, caudal **E** phallus, lateral **F** phallus, dorsal **G** subphallic sclerite, caudal.

Genitalia as in Fig. 1. Sternum IX in lateral view subtriangular, about 2/3 height of segment VIII; in ventral view quadrate, anterior corners sharply rounded, sides slightly constricted mesally, anterior margin moderately concave, posterior margin produced medially. Terga IX + X membranous. Intermediate appendage slightly sinuate, very long, length greater than height of abdomen, basal region turned laterad at base; in dorsal view nearly uniform in diameter throughout length, gradually narrowing apically. Mesolateral process of preanal appendage moderately long, digitate, apex rounded, at base narrowly joined to dorsal portion of mesoventral process; mesoventral process directed caudad, digitate, about 2/3 length of mesolateral process. Inferior appendage in lateral view short, somewhat triangular; posteroventral margin acute below moderate caudal emargination; dorsolateral flange relatively high, rounded dorsally, apically tapered to rounded or sharp point, with prominent apicoventral point, exposed in lateral view; mesoventral spine present, narrow, in lateral view acute, positioned medially; in ventral view inferior appendage broad basally, slender, tapering apically, caudomesal spine prominent, acute; mesoventral spine hidden. Phallobase short; in lateral view apicoventral projection narrow, slightly longer than apical diameter of phallobase apex, with 2 points; separated by shallow median groove; endothecal sclerotic band somewhat broad, becoming less sclerotized apically; endothecal spines absent; phallotremal sclerite narrow in dorsal aspect. Subphallic sclerite Y-shaped, arms long, pedicel with narrow lateral expansions; narrow in lateral view, ventrally somewhat narrowed.

#### Holotype male:

**BRAZIL: São Paulo:** Estação Biológica Boraceia, Riberão Coruja, 2.iv.1977, C.M. & O.S. Flint, Jr. (UMSP000136631) (NMNH).

#### Paratypes:

same data as holotype, 7 males (NMNH); Res. Casa Grande, Pedreira, 13.x.1974, Froehlich, 1 male (in alcohol) (NMNH); Res. Casa Grande, Riberão Coruja, 26.i.1974, Froehlich, 1 male, 1 female (in alcohol) (NMNH); Est. Biol. Paranapiacaba, 6.viii. 1963, Froehlich, 1 male (in alcohol) (NMNH); same, except 27.viii.1963, 1 male (in alcohol) (NMNH); Estação Biológica Boraceia, Rio Guaratuba, 23°40.039'S, 45°53.759'W, 775 m, 17.iv.1998, Holzenthal, Melo, Froehlich, 1 male (UMSP); same, except 17.ix.2002, Blahnik, Prather, Melo, Froehlich, Silva, 1 male (UMSP).

#### Etymology.

Named for Estação Biológica Boraceia, the biological station located on the forested slopes of the Serra do Mar, where the holotype was collected.

### Polycentropus galharada

Hamilton & Holzenthal sp. n.

urn:lsid:zoobank.org:act:BF3019BD-3B2D-44FE-B25E-27FB65FBA33E

Fig. 2 

#### Description.

As noted in the diagnosis of Polycentropus boraceia, this new species and the other 5 new species of the *urubici* cluster are similar to, but distinct from Polycentropus urubici based on the shape and length of the intermediate appendage as well as the shape of the inferior appendage. Separated from Polycentropus boraceia as noted above, Polycentropus galharada sp. n. also resembles Polycentropus froehlichi sp. n., Polycentropus ancistrus sp. n., and Polycentropus graciosa sp. n. in the shape of the inferior appendage. It is distinguished from these species in details of this organ, particularly in the shape of the dorsolateral flange and the caudomesal spine in addition to the shapes and relative lengths of the processes of the preanal appendage.

##### Adult.

Length of forewing (male) 7.5–8 mm. Body dark brown to black; dorsum of head and thorax black, clothed with long, erect dark setae; base of forewing with long, erect black setae, general vestiture of forewing with fine black setae, lacking patches of pale setae; legs dark brown to black.

##### Male.

**Figure 2.**
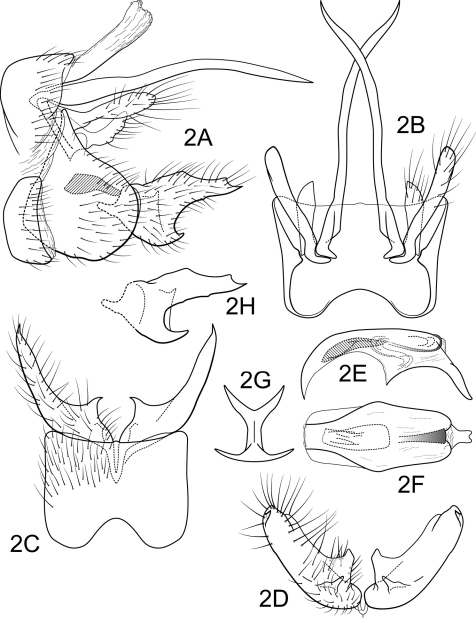
Polycentropus galharada sp. n. Male genitalia: **A** lateral **B** dorsal **C** ventral **D** inferior appendages, caudal **E** phallus, lateral **F** phallus, dorsal **G** subphallic sclerite, caudal **H** inferior appendage, variant, lateral.

Genitalia as in Fig. 2. Sternum IX in lateral view broadly subtriangular, about 3/4 height of segment VIII; in ventral view quadrate, anterior corners very broadly rounded, sides straight, anterior margin deeply concave, posterior margin slightly concave. Terga IX + X membranous, slightly sclerotized ventrally. Intermediate appendage gently curving ventromesad, very long, length much greater than height of abdomen, basal region slightly expanded; in dorsal view nearly uniform in diameter throughout length, gradually narrowing apically. Mesolateral process of preanal appendage moderately long, digitate, apex somewhat swollen, rounded, at base narrowly joined to dorsal portion of mesoventral process; mesoventral process directed dorsocaudad, digitate, about 2/3 length of mesolateral process. Inferior appendage in lateral view moderately long, somewhat triangular; posteroventral margin acute below moderate (Fig. 2A) to deep (Fig. 2H) caudal emargination; dorsolateral flange low, somewhat straight with 2 shallow excavations of dorsal margin beyond midlength, apically tapered to sharp point, with prominent apicoventral point, exposed in lateral view; mesoventral spine present, narrow, in lateral view acute, positioned medially; in ventral view inferior appendage broad basally, slender, tapering apically, caudomesal spine prominent, acute; mesoventral spine hidden; apex angularly truncate. Phallobase moderately short; in lateral view apicoventral projection narrow, much longer than apical diameter of phallobase apex, with 2 points; separated by shallow median groove; endothecal sclerotic band somewhat broad, becoming less sclerotized apically; endothecal spines absent; phallotremal sclerite narrow in dorsal aspect. Subphallic sclerite Y-shaped, arms long, pedicel with broad lateral expansions; broad in lateral view, ventrally narrowed.

#### Holotype male:

**BRAZIL: São Paulo:** Parque Estadual de Campos do Jordão, Rio Galharada, 22°41.662'S, 45°27.783'W, 1530 m, 22.i.1998, Holzenthal, Froehlich, Paprocki (UMSP000033083) (MZUSP).

#### Paratypes:

**BRAZIL: São Paulo:** Parque Estadual de Campos do Jordão, Cachoeira Galharada, 22°41.735'S, 45°27.725'W, 1620 m, 7.iii.1996, Holzenthal, Rochetti, Oliveira, 4 males, 1 female (UMSP); same, 15.x.1998, Paprocki & Froehlich, 1 male, 2 females (UMSP); Parque Estadual de Campos do Jordão, Rio Galharada, 22°41.662'S, 45°27.783'W, 1530 m, 4–5.iii.1996, Holzenthal & Guahyba, 3 males (UFRJ); same, 22.i.1998, Holzenthal, Froehlich, Paprocki, 2 males (UFBA); Parque Estadual de Campos do Jordão, Campo do Meio, 22°41.750'S, 45°29.448'W, 1500 m, 6.iii.1996, Holzenthal & Guahyba, 3 males (UMSP); same, 21.i.1998, Holzenthal, Froehlich, Paprocki, 1 male, 1 female (PUCMG); Rio Casquilho, 3.4 km NE Parque Estadual Campos do Jordão, 22°40.29'S, 45°27.87'W, 1550 m, 23.i.1998, Holzenthal, Froehlich, Paprocki, 2 males (NMNH); Serra do Japi, Córrego da Ermida and small dam, 23°12'S, 47°00'W, 9–10.xii.1997, Froehlich, 1 male (MZUSP).

#### Etymology.

Named for the type locality.

### Polycentropus froehlichi

Hamilton & Holzenthal sp. n.

urn:lsid:zoobank.org:act:AFA47C3D-3C4E-458E-9EDE-515F63157527

Fig. 3 

#### Description.

Resembling Polycentropus galharada mainly in the shape of the preanal and inferior appendages, Polycentropus froehlichi sp. n. is distinguished from that species and the other species of the *urubici* cluster particularly by the phallobase where the apicoventral process is relatively thick at its base and is perpendicular to the axis of phallobase, when viewed laterally. Also, the base of the mesoventral process of the preanal appendage of Polycentropus froehlichi sp. n. is much broader than that of Polycentropus galharada.

##### Adult.

Length of forewing (male) 7–7.5 mm. Body black; dorsum of head and thorax black, clothed with long, black setae; base of forewing with long, erect black setae, general vestiture of forewing with fine black setae, lacking patches of pale setae; legs dark brown to black.

##### Male.

**Figure 3.**
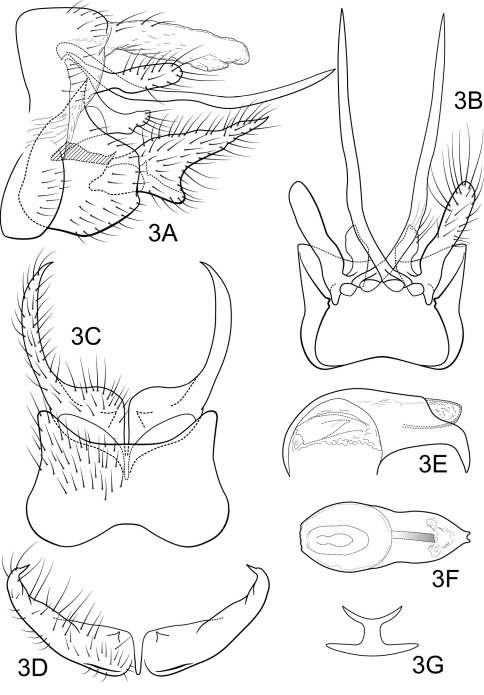
Polycentropus froehlichi sp. n. Male genitalia: **A** lateral **B** dorsal **C** ventral **D** inferior appendages, caudal **E** phallus, lateral **F** phallus, dorsal **G** subphallic sclerite, caudal.

Genitalia as in Fig. 3. Sternum IX in lateral view broadly subtriangular, about 2/3 height of segment VIII; in ventral view slightly trapezoidal, anterior corners very broadly rounded, sides strongly constricted mesally, anterior margin moderately concave, posterior margin deeply and broadly concave. Terga IX + X membranous, slightly sclerotized ventrally; with several long, slender setae. Intermediate appendage slightly curved dorsad, very long, length much greater than height of abdomen, basal region slightly expanded; in dorsal view nearly uniform in diameter throughout length, gradually narrowing apically. Mesolateral process of preanal appendage moderately long, broadly digitate, apex rounded, at base narrowly joined to dorsal portion of mesoventral process; mesoventral process directed caudad, very broad basally, narrowing to irregularly rounded apex, about 1/2 length of mesolateral process. Inferior appendage in lateral view moderately long, somewhat triangular; posteroventral margin protruding, rounded below shallow caudal emargination; dorsolateral flange low, slightly rounded dorsally, apically tapered to sharp inturned point, without caudomesal spine; mesoventral spine present, broad, in lateral view obtuse, positioned medially; in ventral view inferior appendage broad basally, slender, tapering apically; mesoventral spine hidden; apex acute. Phallobase short; in lateral view apicoventral projection moderately broad, slightly shorter than diameter of apical diameter of phallobase apex, with 2 points; separated by shallow median groove; endothecal sclerotic band somewhat broad, becoming less sclerotized apically; phallotremal sclerite wide in dorsal aspect. Subphallic sclerite Y-shaped, arms short, pedicel with narrow lateral expansions; broad in lateral view, ventrally somewhat narrowed.

#### Holotype male:

**BRAZIL: Santa Catarina:** Morro da Igreja, Cachoeira Veu da Noiva, 28°04.595'S, 49°31.090'W, 1300 m, 5.iii.1998, Holzenthal, Froehlich, Paprocki (UMSP000033101) (MZUSP).

#### Paratypes:

**BRAZIL: Santa Catarina:** Urubici, Cachoeira Avencal, 28°02.839'S, 49°36.997'W, 1260 m, 6.iii.1998, Holzenthal, Froehlich, Paprocki, 1 male, 1 female (UMSP); same data as holotype, 1 male, 1 female (UMSP).

#### Etymology.

Named for the great Brazilian aquatic entomologist, Dr. Claudio G. Froehlich, University of São Paulo, in recognition of his lifelong study of the aquatic insects of Brazil.

### Polycentropus ancistrus

Hamilton & Holzenthal sp. n.

urn:lsid:zoobank.org:act:57255A7B-5BC9-4FE0-95B7-472D92952D73

Fig. 4. 

#### Description.

Polycentropus ancistrus sp. n. most closely resembles Polycentropus froehlichi. It is distinguished from Polycentropus froehlichi and the other *urubici* cluster species by the strongly incurved and acute apices of the dorsolateral flange and caudomesal spine of the inferior appendage as well as the near equal length of both processes of the preanal appendage.

##### Adult.

Length of forewing (male) 6 mm. Body brown; dorsum of head and thorax brown; legs stramineous.

##### Male.

**Figure 4.**
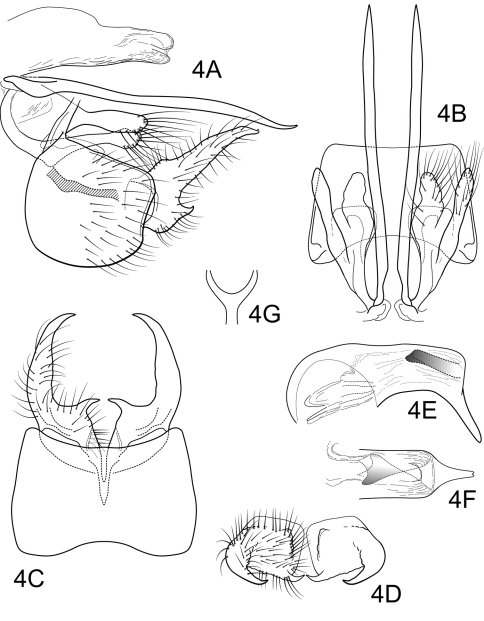
Polycentropus ancistrus sp. n. Male genitalia: **A** lateral **B** dorsal **C** ventral **D** inferior appendages, caudal **E** phallus, lateral **F** phallus, dorsal **G** subphallic sclerite, caudal.

Genitalia as in Fig. 4. Sternum IX in lateral view nearly round, slightly greater than 1/2 height of segment VIII; in ventral view slightly trapezoidal, anterior corners very sharply rounded, sides slightly convex, narrowed posteriorly, anterior margin shallowly concave, posterior margin shallowly concave. Terga IX + X membranous. Intermediate appendage straight, very long, length much greater than height of abdomen, basal region simple, not expanded; in dorsal view nearly uniform in diameter throughout length, gradually narrowing apically. Mesolateral process of preanal appendage moderately long, digitate, apex rounded, at base broadly joined to dorsal 1/2 of mesoventral process; mesoventral process directed caudad, size and shape of mesolateral process, slightly shorter than length of mesolateral process. Inferior appendage in lateral view moderately long, somewhat triangular; posteroventral margin acute below shallow caudal emargination; dorsolateral flange low, slightly excavated medially, apically tapered to sharp inturned point, with prominent caudomesal spine, exposed in lateral view; mesoventral spine absent; in ventral view inferior appendage broad basally, slender, tapering apically, caudomesal spine prominent, acute. Phallobase moderately short; in lateral view apicoventral projection narrow, slightly longer than apical diameter of phallobase apex, with 2 points; separated by very shallow median groove; endothecal sclerotic band forming flanges within phallobase; phallotremal sclerite difficult to discern due to specimen condition. Subphallic sclerite Y-shaped, arms long, pedicel narrow; narrow in lateral view, ventrally somewhat narrowed.

#### Holotype male:

**BRAZIL: São Paulo:** Res. Casa Grande, Rib. Courja, á luz, 26.i.1974, Froehlich, (UMSP000131229) (in alcohol) (MZUSP).

#### Etymology.

From the Greek *ankistron* for fishhook, in reference to the caudomesal spines on the inferior appendage of the male genitalia.

### Polycentropus graciosa

Hamilton & Holzenthal sp. n.

urn:lsid:zoobank.org:act:98D3E5AB-723B-48BE-8184-ED084D0D5545

Fig. 5 

#### Description.

Among the 6 new species in the *urubici* cluster, Polycentropus graciosa sp. n. resembles Polycentropus boraceia, Polycentropus galharada, Polycentropus froehlichi, and Polycentropus ancistrus in the shape of the inferior appendage with the elongate dorsolateral flange. In Polycentropus graciosa sp. n.,the dorsolateral flange of the inferior appendage is uniformly wide in lateral view and narrows relatively abruptly to a rounded or acute apex. Likewise, the caudomesal spine of the inferior appendage is absent, forming a rounded protrusion, and the mesoventral spine in posterior aspect is bifurcate unlike any of the aforementioned species.

##### Adult.

Length of forewing (male) 7–8 mm. Body dark brown to black; dorsum of head and thorax black, clothed with long, black setae; base of forewing with long, erect black setae, general vestiture of forewing with fine black setae, lacking patches of pale setae; legs dark brown to black.

##### Male.

**Figure 5.**
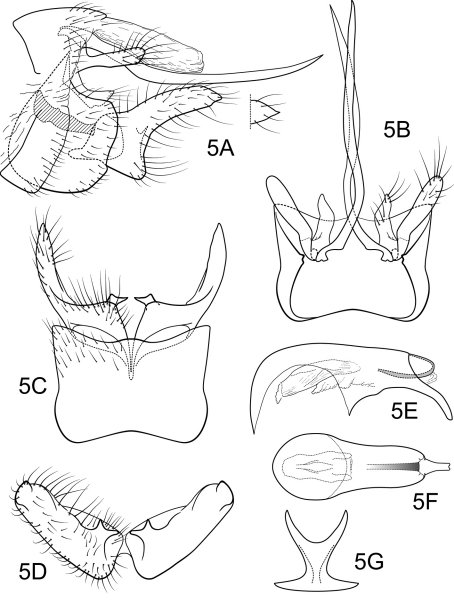
Polycentropus graciosa sp. n. Male genitalia: **A** lateral (inset, variant of inferior appendage apex) **B** dorsal **C** ventral **D** inferior appendages, caudal **E** phallus, lateral **F** phallus, dorsal **G** subphallic sclerite, caudal.

Genitalia as in Fig. 5. Sternum IX in lateral view broadly subtriangular, about 3/4 height of segment VIII; in ventral view quadrate, anterior corners sharply rounded, sides slightly constricted mesally, anterior margin shallowly concave, posterior margin moderately concave with small, shallow convex medial region. Terga IX + X membranous. Intermediate appendage slightly curved dorsad, very long, length much greater than height of abdomen, basal region slightly expanded; in dorsal view nearly uniform in diameter throughout length, gradually narrowing apically. Mesolateral process of preanal appendage moderately long, digitate, apex roundly truncate, at base narrowly joined to dorsal portion of mesoventral process; mesoventral process directed caudad, broadly digitate, about 2/3 length of mesolateral process. Inferior appendage in lateral view moderately long, somewhat triangular; posteroventral margin protruding, rounded below shallow caudal emargination; dorsolateral flange low, slightly rounded dorsally, apically tapered to rounded point, without caudomesal spine; mesoventral spine present, broad, in lateral view acute, bifurcate, positioned well caudad; in ventral view inferior appendage broad basally, slender, tapering apically; mesoventral spine prominent; apex acute, bifurcate. Phallobase moderately short; in lateral view apicoventral projection narrow, approximately equal to apical diameter of phallobase apex, with 2 points; separated by shallow median groove; endothecal sclerotic band somewhat broad, becoming less sclerotized apically; endothecal spines absent; phallotremal sclerite wide in dorsal aspect. Subphallic sclerite U-shaped, arms long, pedicel with narrow lateral expansions; broad in lateral view, ventrally somewhat narrowed.

#### Holotype male:

**BRAZIL: Paraná:** Rio Cascata, Graciosa, road to Morretes, 25°20.214'S, 48°53.971'W, 750 m, 10.i.1998, Holzenthal, Melo, Almeida (UMSP000033088) (MZUSP).

#### Paratypes:

same data as holotype, 2 males, 1 female (UMSP), 1 female (MZUSP); **Paraná:** Quatro Barras, 900 m, 31.i.1993, V.O. Becker, 3 males, 2 females (NMNH).

#### Etymology.

Named for the type locality, a river flowing through the lush Atlantic forest of the Serra da Graciosa, Paraná, Brazil.

### Polycentropus carioca

Hamilton & Holzenthal sp. n.

urn:lsid:zoobank.org:act:DDDEDD30-FF55-4D22-9D16-0D336CC94874

Fig. 6 

#### Description.

Polycentropus carioca sp. n. is the most unusual of the species in the *urubici* cluster. In lateral view, the mesoventral process of the preanal appendage is nearly as wide as long, while in the other 6 species this process is always much narrower relative to its length. Also, in lateral view the inferior appendage in Polycentropus carioca sp. n.is short with the dorsolateral flange being compacted and rounded while the mesoventral spine in posterior view is large and erect.

##### Adult.

Adult. Length of forewing (male) 7 mm. Body dark brown; dorsum of head and thorax dark brown, clothed with long, erect dark setae; base of forewing with long, erect dark setae, general vestiture of forewing with fine black setae, lacking patches of pale setae; legs dark brown to black.

##### Male.

**Figure 6.**
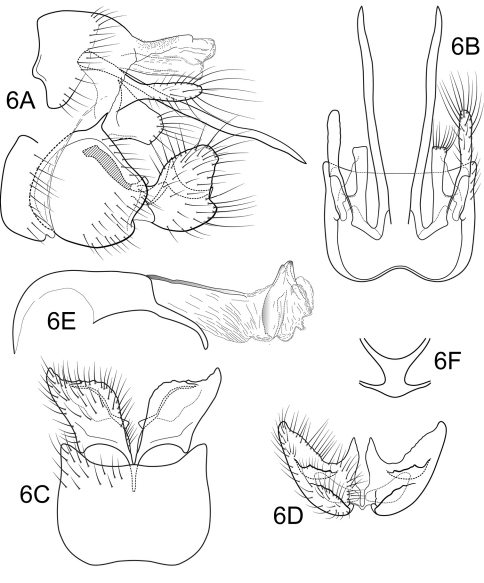
Polycentropus carioca sp. n. Male genitalia: **A** lateral **B** dorsal **C** ventral **D** inferior appendages, caudal **E** phallus, lateral **F** subphallic sclerite, caudal.

Male. Genitalia as in Fig. 6. Sternum IX in lateral view nearly round, approximately 1/2 height of segment VIII; in ventral view quadrate, anterior corners very broadly rounded, sides slightly constricted posteriorly, posterior margin moderately concave with small, shallow convex medial region. Terga IX + X mostly membranous with light dorsal sclerotization basally; with numerous scattered minute spicules. Intermediate appendage straight, very long, length greater than height of abdomen, basal region simple, not expanded; in dorsal view nearly uniform in diameter throughout length, gradually narrowing apically. Mesolateral process of preanal appendage moderately long, digitate, apex rounded, at base narrowly joined to dorsal portion of mesoventral process; mesoventral process directed ventrocaudad, broadly truncate, about 2/3 length of mesolateral process. Inferior appendage in lateral view short, somewhat triangular; posteroventral margin protruding, rounded below shallow caudal emargination; dorsolateral flange relatively high, rounded dorsally, without caudomesal spine; mesoventral spine large, erect, narrow, in lateral view acute, positioned more basad; in ventral view inferior appendage rhomboidal, broadest apically; mesoventral spine with apex visible. Phallobase moderately short; much longer than apical diameter of phallobase apex, with 1 point; endothecal sclerotic band narrow, becoming less sclerotized apically; endothecal spines absent; phallotremal sclerite narrow in dorsal aspect. Subphallic sclerite Y-shaped, arms long, pedicel with broad lateral expansions; narrow in lateral view, ventrally somewhat narrowed.

#### Holotype male:

**BRAZIL: Rio de Janeiro:** Parque Nacional da Serra dos Órgãos, Rio Beija-flor, 22°27.063'S, 43°00.065'W, 1125 m, 27.ii.2002, Holzenthal, Blahnik, Paprocki, Prather (UMSP000136602) (MZUSP).

#### Paratype:

same data as holotype, 1 female (MZUSP).

#### Etymology.

*Carioca* is the Portuguese demonym for the inhabitants of the city of Rio de Janeiro.

### Polycentropus fluminensis

Hamilton & Holzenthal sp. n.

urn:lsid:zoobank.org:act:A16AC0C6-F467-4EA0-90BB-004170F7F224

Fig. 7

Polycentropus  new species 1 Hamilton 1986: 85–86, 198; Fig. 6.4.

#### Description.

Polycentropus fluminensis sp. n., and the other 9 species of the *aguyje* cluster show similarities in the shape of both processes of the preanal appendage, the strongly, often decurved intermediate appendage and the general appearance of the inferior appendage. Among the 10 species of this cluster, Polycentropus fluminensis sp. n.has the shortest and most compact inferior appendage with a strongly reduced dorsolateral plane and the mesoventral process of the preanal appendage is notably broad and short, barely exceeding the mesoventral process.

##### Adult.

Length of forewing (male) 6.5–7.5 mm. Body dark brown to black; dorsum of head and thorax black, clothed with long, black setae; base of forewing with long, erect black setae, general vestiture of forewing with fine black setae, lacking patches of pale setae; legs dark brown to black.

##### Male.

**Figure 7.**
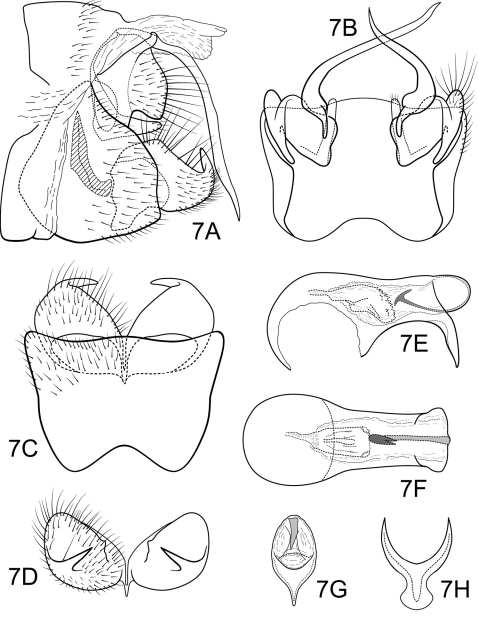
Polycentropus fluminensis sp. n. Male genitalia: **A** lateral **B** dorsal **C** ventral **D** inferior appendages, caudal **E** phallus, lateral **F** phallus, dorsal **G** phallobase, caudal **H** subphallic sclerite, caudal.

Genitalia as in Fig. 7. Sternum IX in lateral view broadly subtriangular, about 2/3 height of segment VIII; in ventral view slightly trapezoidal, anterior corners sharply rounded, sides very slightly constricted mesally, anterior margin deeply concave, posterior margin slightly concave with broad, shallow convex medial region. Terga IX + X membranous. Intermediate appendage gently curving ventromesad, long, length slightly greater than height of abdomen, basal region slightly expanded; in dorsal view nearly uniform in diameter throughout length, gradually narrowing apically. Mesolateral process of preanal appendage short, apex rounded, with slight apicoventral extension, at base broadly joined to mesoventral process; mesoventral process directed caudad, digitate, equal in length to mesolateral process. Inferior appendage in lateral view very short, generally oval; dorsolateral flange very low, rounded dorsally, with prominent caudomesal spine, exposed in lateral view; mesoventral spine present, broad, in lateral view rounded, positioned well basad; in ventral view inferior appendage approximately oval, caudomesal spine prominent, acute. Phallobase moderately short; in lateral view apicoventral projection narrow, slightly longer than apical diameter of phallobase apex, with 1 point; endothecal sclerotic band narrow, ending in pair of small spines resembling a claw-hammer; endothecal spines absent; phallotremal sclerite wide in dorsal aspect. Subphallic sclerite Y-shaped, arms long, pedicel with broad lateral expansions; narrow in lateral view, ventrally somewhat broadened.

#### Holotype male:

**BRAZIL: Rio de Janeiro:** km 17, 18 km S of Teresopolis, 1180 m, 18–19.iv.1977, C.M. & O.S. Flint, Jr. (UMSP000136606) (NMNH).

#### Paratypes:

same data as holotype, 5 males, 2 females (NMNH); **Minas Gerais:** Parque Nacional do Caparaó, small trib to Rio Caparaó, Vale Verde, 20°25.029'S, 41°50.767'W, 1350 m, 12–14.iii.2002, Holzenthal, 1 male (in alcohol) (UMSP); **Rio de Janeiro:** Parque Nacional da Serra dos Órgãos, Rio Paquequer, 22°26.992'S, 42°59.899'W, 1000 m, 26.ii.2002, Holzenthal, Blahnik, Paprocki, Prather, 1 male (UMSP).

#### Etymology.

The word *fluminensis* is derived from the Portuguese demonym for the inhabitants of the state of Rio de Janeiro.

### Polycentropus tripui

Hamilton & Holzenthal sp. n.

urn:lsid:zoobank.org:act:225395E4-9CDB-4227-9E44-7C1083734B80

Fig. 8 

#### Description.

In lateral aspect, this species most closely resembles Polycentropus fluminensis, in the compactness of the inferior appendage and the breadth of the mesolateral process of the preanal appendage. In Polycentropus tripui sp. n., the dorsolateral flange of inferior appendage is larger and the mesoventral process of the preanal appendage is much broader in lateral aspect. The phallic apparatus provides several unique characters that separate this species from the other species described in this paper. The phallobase is very short and there is a very large U-shaped sclerite at the apex of the sclerotized band of the phallic membrane (typically found folded back within the phallobase).

##### Adult.

Length of forewing (male) 6–6.5 mm. Body brown to dark brown; dorsum of head and thorax dark brown, clothed with long, erect dark setae; base of forewing with long, erect dark setae, general vestiture of forewing with fine brown setae, lacking patches of pale setae (in alcohol); legs brown.

##### Male.

**Figure 8.**
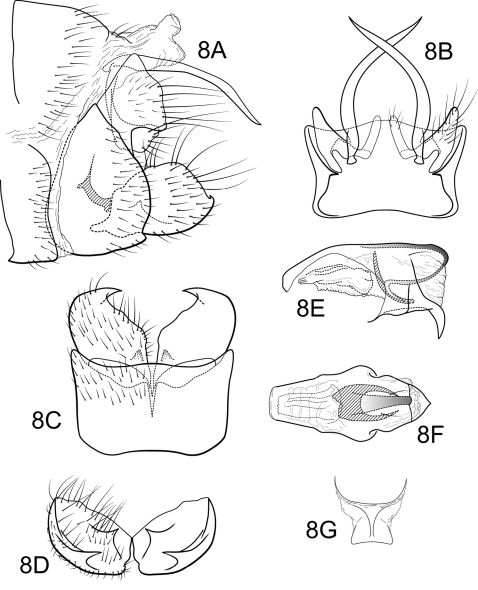
Polycentropus tripui sp. n. Male genitalia: **A** lateral **B** dorsal **C** ventral **D** inferior appendages, caudal **E** phallus, lateral **F** phallus, dorsal **G** subphallic sclerite, caudal.

Genitalia as in Fig. 8. Sternum IX in lateral view subtriangular, about 2/3 height of segment VIII; in ventral view quadrate, anterior corners very broadly rounded, sides very slightly constricted mesally, anterior margin shallowly concave, posterior margin slightly concave with very broad, shallow convex medial region. Terga IX + X membranous. Intermediate appendage gently curving ventromesad, moderate elongate, length about two-thirds height of sternum IX, basal region simple, not expanded; in dorsal view nearly uniform in diameter throughout length, gradually narrowing apically. Mesolateral process of preanal appendage short, apex truncate, with slight apicoventral extension, at base broadly joined to mesoventral process; mesoventral process directed caudad, broad, hatch-shaped, slightly shorter than length of mesolateral process. Inferior appendage in lateral view short, generally oval; dorsolateral flange very low, rounded dorsally, with prominent caudomesal spine, slightly exposed in lateral view; mesoventral spine present, narrow, in lateral view acute, positioned well basad; in ventral view inferior appendage approximately oval, caudomesal spine prominent, acute; mesoventral spine hidden. Phallobase very short; in lateral view apicoventral projection narrow, much shorter than diameter of apical diameter of phallobase apex, with 2 points; endothecal sclerotic band broad, very large hooked process, appearing U-shaped in dorsal view; endothecal spines absent; phallotremal sclerite wide in dorsal aspect. Subphallic sclerite Y-shaped, arms short, pedicel with broad lateral expansions; broad in lateral view, ventrally somewhat broadened.

#### Holotype male:

**BRAZIL: Minas Gerais:** Estação Ecológica de Tripuí, Córrego Tiririca, 20°23.009'S, 43°33.237'W, 19.ii.1999, Paprocki, Amarante, Salgado (UMSP000046917) (MZUSP).

#### Paratypes:

**BRAZIL: Minas Gerais:** Two males (UMSP); same data as holotype, Estação Ecológica de Tripuí, Córrego Botafogo, 20°22.908'S, 43°33.615'W, 1100 m, 25.xi.2001, Holzenthal, Paprocki, Blahnik, Neto, 1 male (UMSP); Estação Ecológica do Tripuí, Córrego Tripuí, 20°23.364'S, 43°32.541'W, 1070 m, 1.xi.1998, Paprocki, Braga, Amarante, 1 male (UFRJ); same, except 21.xi.1998, Paprocki, Braga, Amarante, 1 male (MZUSP); Cachoeira do Abacaxi, Vale do Tropeiro, Ouro Preto, 20°12.270'S, 43°38.163'W, 1120 m, 7.xi.2001, Holzenthal, Paprocki, Blahnik, Amarante, 1 male (UFBA).

#### Etymology.

Named for the small stream in the ecological station of the same name, known for harboring an endemic species of onychophoran, Peripatus acacioi Marcus and Marcus. *Tripui* is the indigenous Tupi-Guarani word for fast or quick water.

### Polycentropus soniae

Hamilton & Holzenthal sp. n.

urn:lsid:zoobank.org:act:34524969-D2DC-4AD6-94A7-6523CBC47433

Fig. 9 

#### Description.

Polycentropus soniae sp. n. most closely resembles Polycentropus fluminensis and Polycentropus tripui, particularly in the ventral aspect of the compact inferior appendage. It can be separated from these species by the angular dorsolateral flange of the inferior appendage and the narrower and rounded mesolateral process of the preanal appendage.

##### Adult.

Length of forewing (male) 6–7 mm. Body dark brown to black; dorsum of head and thorax black, clothed with long, erect dark setae; base of forewing with long, erect black setae, general vestiture of forewing with fine black setae, lacking patches of pale setae; legs dark brown to black.

##### Male.

**Figure 9.**
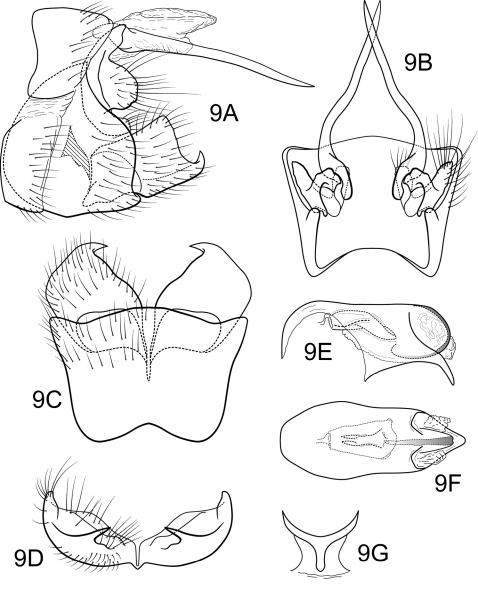
Polycentropus soniae sp. n. Male genitalia: **A** lateral **B** dorsal **C** ventral **D** inferior appendages, caudal **E** phallus, lateral **F** phallus, dorsal **G** subphallic sclerite, caudal.

Genitalia as in Fig. 9. Sternum IX in lateral view broadly subtriangular, about 3/4 height of segment VIII; in ventral view trapezoidal, anterior corners broadly rounded, sides very slightly constricted mesally, anteriorly, posterior margin slightly concave with very broad, shallow convex medial region. Terga IX + X membranous. Intermediate appendage slightly curved on basal 1/3 and relatively straight for remainder of length, long, length slightly greater than height of abdomen, basal region slightly expanded; in dorsal view nearly uniform in diameter throughout length, gradually narrowing apically. Mesolateral process of preanal appendage very short, apex rounded, at base broadly joined to ventral 2/3 of mesoventral process; mesoventral process directed caudad, broadly digitate, slightly shorter than length of mesolateral process. Inferior appendage in lateral view short, quadrate; dorsolateral flange low, straight dorsally, with prominent caudomesal spine, exposed in lateral view; mesoventral spine present, broad, in lateral view rounded, positioned more basad; in ventral view inferior appendage approximately oval, caudomesal spine prominent, acute; mesoventral spine hidden. Phallobase short; in lateral view apicoventral projection narrow, much shorter than diameter of apical diameter of phallobase apex, with 1 point; endothecal sclerotic band narrow, becoming rapidly less sclerotized toward apex; endothecal spines absent; phallotremal sclerite wide in dorsal aspect. Subphallic sclerite Y-shaped, arms long, pedicel with broad lateral expansions; broad in lateral view, ventrally narrowed.

#### Holotype male:

**BRAZIL: Paraná:** Rio Mãe Catira, 10 km N Porto de Cima, 25°21.821'S, 48°52.473'W, 200 m, 8–9.xii.1997, Holzenthal & Huisman (UMSP000033175) (MZUSP).

#### Paratypes:

**BRAZIL: Paraná:** same data as holotype, 10 males, 3 females (UFBA), 10 males, 3 females (UFRJ), 10 males, 3 females (MZUSP), 10 males, 2 females (NMNH), 20 males, 6 females (UMSP); trib. to Rio Mãe Catira, 10.5 km N Porto de Cima, 25°21.778'S, 48°52.590'W, 200 m, 10.xii.1997, Holzenthal & Huisman, 28 males, 2 females (UMSP).

#### Etymology.

Named for Dr. Sonia N. Lazzari, professor of entomology at the Universidade Federal do Paraná, Curitiba, Brazil, in appreciation of her help and friendship during the junior author’s studies in Brazil.

### Polycentropus cheliceratus

Hamilton & Holzenthal sp. n.

urn:lsid:zoobank.org:act:E09DE9A9-2B59-4D75-86B6-03A5EA4EEE81

Fig. 10

Polycentropus  new species 2 Hamilton 1986: 86–87, 199; Fig. 6.5.

#### Description.

Very similar to Polycentropus minero sp. n., Polycentropus cheliceratus sp. n.can be distinguished from that species as well as the other 8 species of the *aguyje* cluster by the shape of the inferior appendage in both lateral and ventral views as well as the lateral aspect of the preanal appendage. Compared to Polycentropus minero sp. n., in lateral view, the caudoventral corner and the dorsoventral flange of the inferior appendage are less angular and the mesoventral spine is absent. Also, in Polycentropus cheliceratus sp. n. the mesolateral process of the preanal appendage is smaller in dorsoventral length and its mesoventral process is more slender and longer compared to the mesolateral process.

##### Adult.

Length of forewing (male) 6.6–7.6 mm. Body dark brown to black; dorsum of head and thorax black, clothed with long, black setae; base of forewing with long, erect black setae, general vestiture of forewing with fine black setae, lacking patches of pale setae; legs brown.

##### Male.

**Figure 10.**
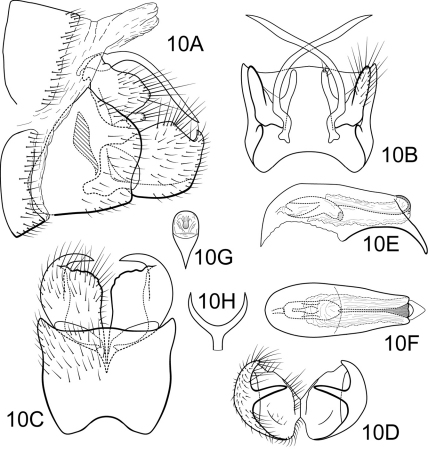
Polycentropus cheliceratus sp. n. Male genitalia: **A** lateral **B** dorsal **C** ventral **D** inferior appendages, caudal **E** phallus, lateral **F** phallus, dorsal **G** phallobase, caudal **H** subphallic sclerite, caudal.

Genitalia as in Fig. 10. Sternum IX in lateral view quadrate, about 2/3 height of segment VIII; in ventral view slightly trapezoidal, anterior corners sharply rounded, sides very slightly constricted mesally, anterior margin deeply concave, posterior margin slightly concave with broad, shallow convex medial region. Terga IX + X membranous, slightly sclerotized ventrally. Intermediate appendage gently curving ventromesad, moderate elongated length about equal to height of sternum IX, basal region simple, not expanded; in dorsal view nearly uniform in diameter throughout length, gradually narrowing apically. Mesolateral process of preanal appendage short, apex rounded, at base broadly joined to mesoventral process; mesoventral process directed ventrad to rounded point, digitate, equal in length to mesolateral process. Inferior appendage in lateral view short, generally round; dorsolateral flange low, rounded dorsally, with prominent caudomesal spine, partially exposed in lateral view; mesoventral spine absent; in ventral view inferior appendage approximately oval, caudomesal spine prominent, acute. Phallobase moderately short; in lateral view apicoventral projection narrow, slightly longer than apical diameter of phallobase apex, with 1 point; endothecal sclerotic band very narrow, becoming less sclerotized apically; endothecal spines absent; phallotremal sclerite narrow in dorsal aspect. Subphallic sclerite Y-shaped, arms long, pedicel narrow in posterior and lateral views.

#### Holotype male:

**BRAZIL: Rio de Janeiro:** km 17, 18 km S of Teresopolis, 1180 m, 18–19.iv.1977, C.M. & O.S. Flint, Jr. (UMSP000136614) (NMNH).

#### Paratypes:

**BRAZIL:** same data as holotype, 1 male (NMNH); **Rio de Janeiro:** Nova Friburgo, municipal water supply, 950 m, 24.iv.1977, C.M. & O.S. Flint, Jr., 3 males, 1 female (NMNH).

#### Etymology.

From the Latin *chela* (Greek *chele*) for claw, in reference to the claw-like form of the inferior appendage of the male genitalia, particularly when viewed in ventral aspect.

### Polycentropus minero

Hamilton & Holzenthal sp. n.

urn:lsid:zoobank.org:act:FE9BF381-404C-4B17-B98E-0699A5F2512D

Fig. 11

Polycentropus  new species 5 Hamilton 1986: 90–91, 202; Fig. 6.8.

#### Description.

Similar to Polycentropus cheliceratus, the more angular shape of the inferior appendage in lateral and ventral aspects as well as the lateral view of the preanal appendage with its larger mesolateral process and shorter, thick mesoventral process distinguishes Polycentropus minero sp. n. from it and other similar species of the *aguyje* cluster.

##### Adult.

Length of forewing (male) 6.4–8.2 mm. Body dark brown; dorsum of head and thorax black; general vestiture of forewing with fine brown setae (in alcohol); legs brown.

##### Male.

**Figure 11.**
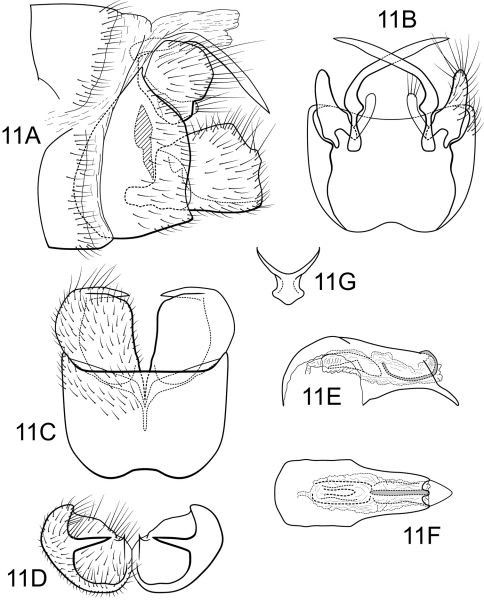
Polycentropus minero sp. n. Male genitalia: **A** lateral **B** dorsal **C** ventral **D** inferior appendages, caudal **E** phallus, lateral **F** phallus, dorsal **G** subphallic sclerite, caudal.

Genitalia as in Fig. 11. Sternum IX in lateral view subtriangular, about 3/4 height of segment VIII; in ventral view quadrate, anterior corners broadly rounded, sides slightly constricted anteriorly, anterior margin shallowly concave, posterior margin shallowly concave. Terga IX + X membranous. Intermediate appendage gently curving ventromesad, moderate elongate, length about two-thirds height of sternum IX, basal region slightly expanded; in dorsal view nearly uniform in diameter throughout length, gradually narrowing apically. Mesolateral process of preanal appendage short, apex rounded, with slight ventral extension, at base broadly joined to mesoventral process; mesoventral process directed ventrad to rounded point, broadly digitate, about 2/3 length of mesolateral process. Inferior appendage in lateral view short, quadrate; dorsolateral flange low, straight dorsally, with prominent caudomesal spine, slightly exposed in lateral view; mesoventral spine present, narrow, in lateral view acute, positioned well basad; in ventral view inferior appendage quadrate, caudomesal spine prominent, acute; mesoventral spine hidden. Phallobase moderately short; in lateral view apicoventral projection narrow, slightly shorter than diameter of apical diameter of phallobase apex, with 1 point; endothecal sclerotic band narrow, becoming less sclerotized apically; endothecal spines absent; phallotremal sclerite narrow in dorsal aspect. Subphallic sclerite Y-shaped, arms long, pedicel with broad lateral expansions; narrow in lateral view, ventrally narrowed.

#### Holotype male:

**BRAZIL: Minas Gerais:** Serra do Cipo, Rio Capivara, 6.vii.1974, Froehlich, Shimizu, et al. (NMNH).

#### Paratypes:

**BRAZIL:** same data as holotype, 1 female (NMNH); same, except 18.xii.1973, Froehlich, 4 males (NMNH); same except 9.ii.1974, Exp. Dep. Zool., 1 male, 2 females (NMNH).

#### Etymology.

The name *minero* is the Portuguese demonym for the inhabitants of Minas Gerais, the Brazilian state where the type specimens were collected.

### Polycentropus carolae

Hamilton & Holzenthal sp. n.

urn:lsid:zoobank.org:act:870EB4ED-EAD0-4CE7-B59F-183A855BE85F

Fig. 12

Polycentropus  new species 3 Hamilton 1986: 87–88, 200; Fig. 6.6.

#### Description.

Polycentropus carolae sp. n. is most similar in many features to Polycentropus minero and Polycentropus caaete sp. n. In Polycentropus carolae sp. n., the mesoventral process of the preanal appendage slightly exceeds the mesolateral process, whereas in Polycentropus minero it is shorter and in Polycentropus caaete sp. n. it is much longer. Also, Polycentropus carolae sp. n.has a shallow excavation in the posterior margin of the inferior appendage while it is absent in Polycentropus minero and deeper in Polycentropus caaete sp. n. Finally, the triangular shape of the mesolateral process of the preanal appendage and the small claw-like structure at the apex of the endothecal sclerotic band are unique to this species in the 10-species *aguyje* cluster.

##### Adult.

Length of forewing (male) 5.4–6.7 mm. Body dark brown to black; general vestiture of forewing with fine black setae, lacking patches of pale setae; legs brown.

##### Male.

**Figure 12.**
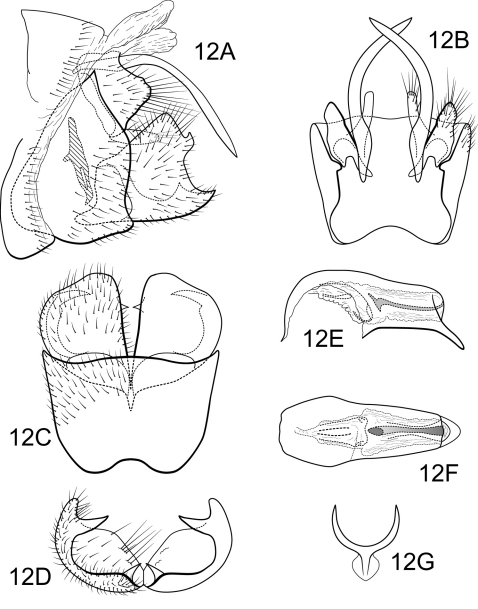
Polycentropus carolae sp. n. Male genitalia: **A** lateral **B** dorsal **C** ventral **D** inferior appendages, caudal **E** phallus, lateral **F** phallus, dorsal **G** subphallic sclerite, caudal.

Genitalia as in Fig. 12. Sternum IX in lateral view subtriangular, about 3/4 height of segment VIII; in ventral view slightly trapezoidal, anterior corners broadly rounded, sides slightly constricted anteriorly, anterior margin moderately concave, posterior margin slightly concave with very broad, shallow convex medial region. Terga IX + X membranous. Intermediate appendage gently curving ventromesad, long, length slightly greater than height of sternum IX, basal region slightly expanded; in dorsal view nearly uniform in diameter throughout length, gradually narrowing apically. Mesolateral process of preanal appendage short, apex slightly triangular, at base broadly joined to mesoventral process; mesoventral process directed caudad, digitate, slightly exceeding length of mesolateral process. Inferior appendage in lateral view short, trapezoidal; posteroventral margin acute below shallow caudal emargination; dorsolateral flange relatively high, straight dorsally, partially exposed in lateral view; mesoventral spine present, broad, in lateral view acute, positioned more caudad; in ventral view inferior appendage approximately round, caudomesal spine hidden; mesoventral spine with apex visible. Phallobase moderately short; in lateral view apicoventral projection narrow, approximately equal to apical diameter of phallobase apex, with 1 point; endothecal sclerotic band narrow, apex terminating in simple claw; endothecal spines absent; phallotremal sclerite wide in dorsal aspect. Subphallic sclerite Y-shaped, arms long, pedicel with broad lateral expansions; narrow in lateral view, ventrally somewhat narrowed.

#### Holotype male:

**BRAZIL: Rio de Janeiro:** km 54, 26 km E Nova Friburgo, 410 m, 19.iv.1977, C.M. & O.S. Flint, Jr. (UMSP000136621) (NMNH).

#### Paratypes:

same data as holotype, 3 males, 1 female; same, except 25.iv.1977, 1 female; Rio de Janeiro: Cachoeiras de Macacu, 800 m, 15.x.1985, Miller, 1 male (NMNH); Parque Nacional da Serra dos Órgãos, Guapimirim, Trilha das Ruínas, 22°29.679'S, 42°59.729'W, 940 m, 28.ii.2002, Blahnik & Paprocki, 2 males (in alcohol) (UMSP).

#### Etymology.

Named with affection for Mrs. Carol Flint in honor of her numerous and important collections of Trichoptera made over many years across Latin America.

### Polycentropus caaete

Hamilton & Holzenthal sp. n.

urn:lsid:zoobank.org:act:B7D82617-4287-44A9-BA7A-87539353276E

Fig. 13 

#### Description.

Polycentropus caaete sp. n. appears to be intermediate in appearance between Polycentropus carolae and Polycentropus itatiaia sp. n. in characters of the inferior appendage. The excavation of its posterior margin is deeper in Polycentropus caaete sp. n., but not as deep as in Polycentropus itatiaia sp. n.; the position of the caudomesal spine is similar to Polycentropus carolae, but it is near the upper apical corner in Polycentropus itatiaia sp. n.; and the mesoventral spine is more basad in Polycentropus caaete sp. n. than in the other 2 species. The relative lengths of the 2 processes of the preanal appendage will also separate Polycentropus caaete sp. n. from the other species. In the thickness of the apicoventral projection of the phallobase and the exposure of the caudomesal spine of the inferior appendage this species bears some resemblance to Polycentropus aguyje but can be separated by other details of the inferior and preanal appendages.

##### Adult.

Length of forewing (male) 6–6.5 mm. Body dark brown to black; dorsum of head and thorax black, clothed with long, erect dark setae; base of forewing with long, erect black setae, general vestiture of forewing with fine black setae, lacking patches of pale setae; legs dark brown to black.

##### Male.

**Figure 13.**
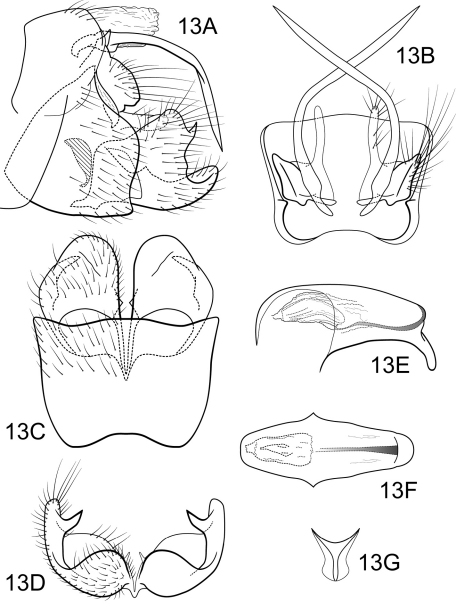
Polycentropus caaete sp. n. Male genitalia: **A** lateral **B** dorsal **C** ventral **D**  inferior appendages, caudal **E** phallus, lateral **F** phallus, dorsal **G** subphallic sclerite, caudal.

Genitalia as in Fig. 13. Sternum IX in lateral view broadly subtriangular, about 2/3 height of segment VIII; in ventral view slightly trapezoidal, anterior corners sharply rounded, sides slightly constricted anteriorly, anterior margin shallowly concave, posterior margin slightly concave with very broad, shallow convex medial region. Terga IX + X membranous. Intermediate appendage long, gently curving ventromesad, length slightly greater than height of abdomen; in dorsal view basal region narrow with slightly expanded segment slightly distad, nearly uniform in diameter throughout length, gradually narrowing apically. Mesolateral process of preanal appendage very short, apex rounded, with slight ventral extension, at base broadly joined to medial portion of mesoventral process; mesoventral process directed caudad, broadly digitate, nearly 2 times the length of mesolateral process. Inferior appendage in lateral view short, quadrate; posteroventral margin rounded, protruding below caudal emargination; dorsolateral flange low, straight dorsally, with prominent caudomesal spine, partially exposed in lateral view; mesoventral spine present, broad, in lateral view acute, positioned more basad; in ventral view inferior appendage approximately oval, caudomesal spine hidden; mesoventral spine with apex visible. Phallobase moderately short; in lateral view apicoventral projection moderately broad, approximately equal to apical diameter of phallobase apex, with 1 point; endothecal sclerotic band narrow, becoming rapidly less sclerotized toward apex; endothecal spines absent; phallotremal sclerite wide in dorsal aspect. Subphallic sclerite Y-shaped, arms short, pedicel with broad lateral expansions; broad in lateral view, ventrally narrowed.

#### Holotype male:

**BRAZIL: Santa Catarina:** Parque Ecológica Spitzkopf, Rio Caeté above 1st falls, 27°00.35'S, 49°06.70'W, 170 m, 26.xi.2003, Holzenthal, Paprocki, Calor (UMSP000120814) (MZUSP).

#### Paratypes:

same data as holotype, 2 males, 2 females (UMSP); **Paraná:** trib. to Rio Mãe Catira, 10.5 km. N Porto de Cima, 25°21.778'S, 48°52.590'W, 200 m, 10.xii.1997, Holzenthal & Huisman, 1 male (UMSP); **São Paulo:** Parque Estadual Intervales, Rio do Carmo, 24°18.983'S, 48°25.250'W, 560 m, 29.ix.2002, Blahnik, Prather, Melo, Calor, 1 male (UFBA); Agua Comprida @ bridge, 24°17.591'S, 48°25.102'W, 600 m, 30.ix.2002, Blahnik, Prather, Melo, Calor, 2 males, 1 female (MZUSP); Estação Biológica de Boracéia, Rio Claro at bridge, 23°39.002'S, 45°54.889'W, 815 m, 19.ix.2002, Blahnik, Prather, Melo, Silva, 1 male (UFRJ); small stream on São Paulo route 247, 11 km SE Bananal, 22°45.684'S, 44°23.190'W, 675 m, 23.ix.2002, Blahnik, Prather, Melo, Froehlich, Silva, 5 males (UMSP).

#### Etymology.

From *caá-etê*, the Tupi-Guarani word for the Atlantic forest of southeastern Brazil.

### Polycentropus itatiaia

Hamilton & Holzenthal sp. n.

urn:lsid:zoobank.org:act:C0F3E691-A6B2-453A-84FE-4C2C9A9A52E5

Fig. 14 

#### Description.

Most similar to Polycentropus aguyje and Polycentropus santateresae sp. n., Polycentropus itatiaia sp. n. differs in the shape and depth of the emargination in the posterior margin of the inferior appendage as well as the median position of the relatively low mesoventral spine of this appendage. The mesolateral process of the preanal appendage in Polycentropus itatiaia sp. n.is triangular, as it is in Polycentropus carolae, and the mesoventral process is shorter and directed more ventrad.

##### Adult.

Length of forewing (male) 6–6.5 mm. Body dark brown; dorsum of head and thorax black, clothed with long, erect dark setae; base of forewing with long, erect black setae, general vestiture of forewing with fine black setae, lacking patches of pale setae; legs dark brown to black.

##### Male.

**Figure 14.**
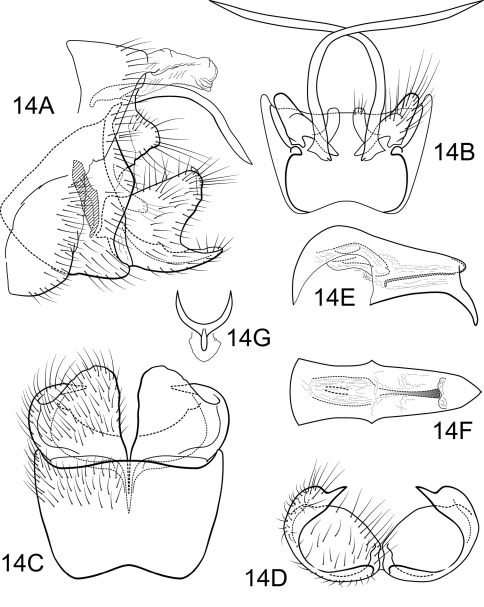
Polycentropus soniae sp. n. Male genitalia: **A** lateral **B** dorsal **C** ventral **D** inferior appendages, caudal **E** phallus, lateral **F** phallus, dorsal **G** subphallic sclerite, caudal.

Genitalia as in Fig. 14. Sternum IX in lateral view subtriangular, about 2/3 height of segment VIII; in ventral view slightly trapezoidal, anterior corners sharply rounded, sides slightly convex, anterior margin shallowly concave, posterior margin slightly concave with very broad, shallow convex medial region. Terga IX + X membranous. Intermediate appendage slightly curved on basal 1/2 and relatively straight for remainder of length, very long, length greater than height of abdomen, basal region slightly expanded; in dorsal view nearly uniform in diameter throughout length, gradually narrowing apically. Mesolateral process of preanal appendage very short, apex slightly triangular, at base broadly joined to medial portion of mesoventral process; mesoventral process directed ventrocaudad, digitate, slightly shorter than length of mesolateral process. Inferior appendage in lateral view short, quadrate; posteroventral margin rounded, protruding below deep caudal emargination; dorsolateral flange low, straight dorsally, with prominent caudomesal spine, slightly exposed in lateral view; mesoventral spine present, broad, in lateral view obtuse, positioned medially; in ventral view inferior appendage approximately round, caudomesal spine partially hidden, acute; mesoventral spine hidden. Phallobase moderately short; in lateral view apicoventral projection narrow, approximately equal to apical diameter of phallobase apex, with 1 point; endothecal sclerotic band narrow, becoming less sclerotized apically; endothecal spines absent; phallotremal sclerite narrow in dorsal aspect. Subphallic sclerite Y-shaped, arms long, pedicel with broad lateral expansions; broad in lateral view, ventrally somewhat narrowed.

#### Holotype male:

**BRAZIL: Rio de Janeiro:** Parque Nacional do Itatiaia, trib. to Rio Taquaral, 22°26.688'S, 44°36.464'W, 1320 m, 6.iii.2002, Holzenthal, Blahnik, Prather (UMSP000136573) (MZUSP).

#### Paratypes:

same data as holotype, 1 female (MZUSP); Parque Nacional do Itatiaia, Rio Campo Belo, trail to Véu da Noiva, 22°25.706'S, 44°37.171'W, 1310 m, 5.iii.2002, Holzenthal, Blahnik, Paprocki, Prather, 1 male, 1 female (UFRJ); Parque Nacional do Itatiaia, Rio Campo Belo, 22°27.033'S, 44°36.818'W, 1300 m, 7.iii.2002, Holzenthal, Blahnik, Paprocki, Prather, 5 males, 2 females (UMSP); **Minas Gerais:** Ibitipoca, sitio of Anestis Papadopolous, 21°43.227'S, 43°54.557'W, 1200 m, 23.x.2000 m, Paprocki, 2 males (UFBA).

#### Etymology.

Named for Parque Nacional Itatiaia where the type specimens were collected. *Itatiaia* means “many-pointed rock” in Tupi-Guarani.

### Polycentropus santateresae

Hamilton & Holzenthal sp. n.

urn:lsid:zoobank.org:act:2FB21067-F8C9-443E-B40E-EBA9931300B1

Fig. 15

Polycentropus  new species 4 Hamilton 1986: 89–90, 201; Fig. 6.7.

#### Description.

**Figure 15.**
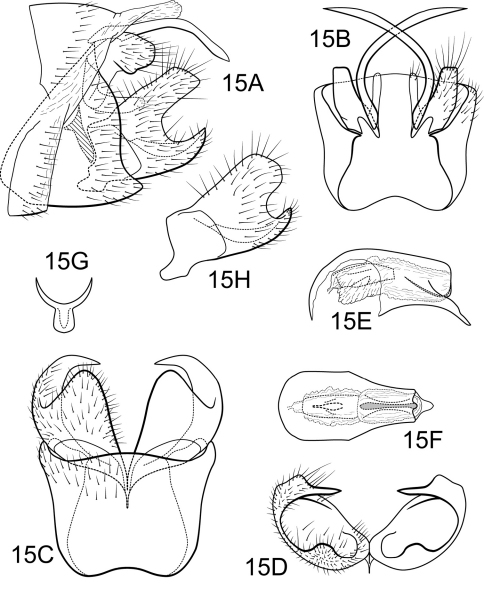
Polycentropus santateresae sp. n. Male genitalia: **A** lateral **B** dorsal **C** ventral **D** inferior appendages, caudal **E** phallus, lateral **F** phallus, dorsal **G** subphallic sclerite, caudal **H** inferior appendage, variant, lateral (Minas Gerais, Rio Caparaó).

Polycentropus santateresae sp. n.is distinct in the shorter length of the intermediate appendage compared to other species of the *aguyje* cluster. The body of the mesolateral process of the preanal appendage is round and positioned on the dorsal half of its base. We have noted some variation in the lateral view of the inferior appendages of the material examined (Fig. 15A and 15H), but at this time we consider this within the range of variation for Polycentropus santateresae sp. n. This species, particularly the variant from Rio Caparaó in Minas Gerais, is most similar to Polycentropus aguyje in the shape of the caudal excavation of the inferior appendage and the shape of the mesolateral process of the preanal appendage. This species can be separated from Polycentropus aguyje based on the narrow shape of apicoventral projection of the phallobase and the hidden position of the caudomesal spine of the inferior appendage in lateral view.

##### Adult.

Length of forewing (male) 5.5–6.5 mm. Body dark brown; dorsum of head and thorax black, clothed with long, erect dark setae; base of forewing with long, erect dark setae, general vestiture of forewing with fine black setae, lacking patches of pale setae; legs brown.

##### Male.

Genitalia as in Fig. 15. Sternum IX in lateral view subtriangular, about 3/4 height of segment VIII; in ventral view trapezoidal, anterior corners broadly rounded, sides very slightly constricted mesally, anterior margin shallowly concave, posterior margin slightly concave with very broad, shallow convex medial region. Terga IX + X membranous. Intermediate appendage gently curving ventromesad, moderate elongate, length slightly less than height of sternum IX, basal region simple, not expanded; in dorsal view nearly uniform in diameter throughout length, gradually narrowing apically. Mesolateral process of preanal appendage short, apex rounded, with slight ventral extension, at base broadly joined to dorsal 1/2 of mesoventral process; mesoventral process directed caudad, digitate, equal in length to mesolateral process. Inferior appendage in lateral view short, quadrate; posteroventral margin acute below deep caudal emargination; dorsolateral flange relatively high, straight dorsally, hidden in lateral view; mesoventral spine present, broad, in lateral view obtuse, positioned more basad; in ventral view inferior appendage quadrate, caudomesal spine prominent, acute; mesoventral spine hidden. Phallobase short; in lateral view apicoventral projection narrow, approximately equal to apical diameter of phallobase apex, with 1 point; endothecal sclerotic band narrow, becoming less sclerotized apically; endothecal spines absent; phallotremal sclerite narrow in dorsal aspect. Subphallic sclerite Y-shaped, arms long, pedicel with broad lateral expansions; narrow in lateral view, ventrally somewhat narrowed.

#### Holotype male:

**BRAZIL: Espirito Santo:** 15 km SE Santa Teresa, Fazenda Santa Clara, 460 m, 22.iv.1977, C.M. & O.S. Flint, Jr. (UMSP000136628) (NMNH)

#### Paratypes:

**BRAZIL: Minas Gerais:** Rio Caparaó, Hotel Parque Caparaó, Alto Caparaó, 20°25.498'S, 41°51.500'W, 830 m, 11–14.iii.2002, Holzenthal, Blahnik, Paprocki, Prather, 1 male (UMSP)

#### Etymology.

Named for the central Espirito Santo town of Santa Teresa near which this species was collected.

### Polycentropus virginiae

Hamilton & Holzenthal sp. n.

urn:lsid:zoobank.org:act:E2F15EC7-EF36-4DA1-BC4D-C44947FE8607

Fig. 16 

#### Description.

Polycentropus virginiae sp. n.is readily distinguished from other *aguyje* cluster species by the shape of the inferior and preanal appendages. Specifically, the prominently visible recurved spine on the inferior appendage as well as the deep excavation below this spine and the large triangular flange lying dorsobasad of the spine are distinct. The mesolateral process of the preanal appendage is broad, low, and round, mostly closely resembling that of only Polycentropus caaete.

##### Adult.

Length of forewing (male) 5.5–7 mm. Body dark brown to black; dorsum of head and thorax black, clothed with long, black setae; base of forewing with long, erect black setae, general vestiture of forewing with fine black setae, lacking patches of pale setae; legs dark brown to black.

##### Male.

**Figure 16.**
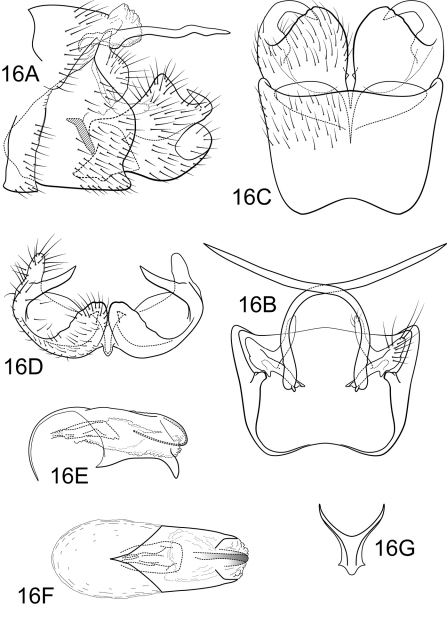
Polycentropus virginiae sp. n. Male genitalia: **A** lateral **B** dorsal **C** ventral **D** inferior appendages, caudal **E** phallus, lateral **F** phallus, dorsal **G** subphallic sclerite, caudal.

Genitalia as in Fig. 16. Sternum IX in lateral view broadly subtriangular, about 3/4 height of segment VIII; in ventral view slightly trapezoidal, anterior corners broadly rounded, sides very slightly constricted mesally, anteriorly, anterior margin moderately concave, posterior margin slightly concave with very broad, shallow convex medial region. Terga IX + X membranous. Intermediate appendage slightly curved on basal 1/2 and relatively straight for remainder of length, very long, length greater than height of abdomen, basal region simple, not expanded; in dorsal view nearly uniform in diameter throughout length, gradually narrowing apically. Mesolateral process of preanal appendage very short, apex rounded, at base broadly joined to mesoventral process; mesoventral process directed caudad, digitate, 2 times the length of mesolateral process. Inferior appendage in lateral view short, trapezoidal; posteroventral margin rounded,protruding below deep caudal emargination; dorsolateral flange relatively high, protruding as rounded triangle, with prominent caudomesal spine, exposed in lateral view; mesoventral spine present, broad, in lateral view obtuse, positioned well basad; in ventral view inferior appendage approximately round, caudomesal spine partially hidden, acute; mesoventral spine with apex visible. Phallobase moderately short; in lateral view apicoventral projection moderately broad, much shorter than diameter of apical diameter of phallobase apex, with 1 point; endothecal sclerotic band narrow, becoming less sclerotized apically; endothecal spines absent; phallotremal sclerite wide in dorsal aspect. Subphallic sclerite Y-shaped, arms long, pedicel with narrow lateral expansions; narrow in lateral view, ventrally somewhat narrowed.

#### Holotype male:

**BRAZIL: Minas Gerais:** Córrego da Serra de Ouro Fino, Vale do Tropeiro, 20°12.371'S, 43°38.581'W, 1000 m, 8.x.2000 m, Paprocki, Salgado, Isaac (UMSP000046828) (MZUSP).

#### Paratypes:

**BRAZIL: Minas Gerais:** Aldeia da Cachoeira das Pedras, 20°06.824'S, 44°01.412'W, 925 m, 28–29.ix.2000 m, Paprocki & Braga, 1 male (UMSP); Cachoeira do Abacaxi, Vale do Tropeiro, Ouro Preto, 20°12.270'S, 43°38.163'W, 1120 m, 7.xi.2001, Holzenthal, Paprocki, Blahnik, Amarante, 1 male (in alcohol) (MZUSP); Rio Caparaó, Hotel Parque Caparaó, Alto Caparaó, 20°25.498'S, 41°51.500'W, 830 m, 11–14.iii.2002, Holzenthal, Blahnik, Paprocki, Prather, 9 males, 3 females (UMSP); Parque Nacional do Caparaó, Rio Caparaó at Vale Verde, 20°25.029'S, 41°50.767'W, 1350 m, 12–13.iii.2002, Holzenthal, Blahnik, Paprocki, Prather, 2 males (UFBA), 2 males (UFRJ), 1 male (NMNH).

#### Etymology.

Named with affection for Virgina Braga, in recognition for her many years of friendship with the junior author and his family.

### Polycentropus cipoensis

Hamilton & Holzenthal sp. n.

urn:lsid:zoobank.org:act:E08F9BE7-0F1E-4E93-97B1-C83F6CA28B7F

Fig. 17

Polycentropus  new species 6 Hamilton 1986: 91–93, 203; Fig. 6.9.

#### Description.

Polycentropus cipoensis sp. n. is clearly a member of the *jorgenseni* species complex, but is otherwise easily separated from the other Neotropical Polycentropus by the inferior appendage which has a triangular base, but extends into a long, narrowed apex and a mesobasal process that is somewhat palmate in its posterior aspect. In addition, the intermediate appendage is short as are the 2 processes of the preanal appendage. The endothecal sclerotic band ends in a pair of prominent hooks, similar to, but larger, than those in Polycentropus fluminensis, Polycentropus tripui, Polycentropus carolae, and Polycentropus acinaciformis sp. n. The apicoventral process of the phallobase, while similar to that seen in other members of the *jorgenseni* species complex, is notably shorter and wider and the apical emargination is broadly u-shape.

##### Adult.

Length of forewing (male) 5.8–7.0 mm. Body brown to dark brown; dorsum of head and thorax dark brown, clothed with long, erect dark setae; base of forewing with long, erect dark setae, general vestiture of forewing with fine brown setae, lacking patches of pale setae (in alcohol); legs brown.

##### Male.

**Figure 17.**
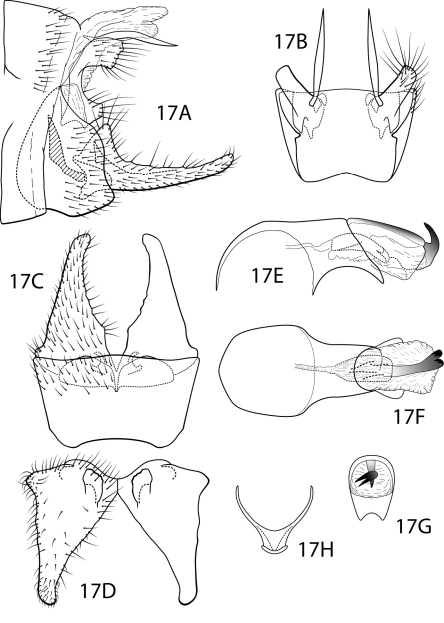
Polycentropus cipoensis sp. n. Male genitalia: **A** lateral **B** dorsal **C** ventral **D** inferior appendages, caudal **E** phallus, lateral **F** phallus, dorsal **G** phallobase, caudal **H** subphallic sclerite, caudal.

Genitalia as in Fig. 17. Sternum IX in lateral view subtriangular, slightly greater than 1/2 height of segment VIII; in ventral view trapezoidal, anterior corners sharply rounded, sides slightly constricted anteriorly, anterior margin shallowly concave, posterior margin slightly concave with broad, shallow convex medial region. Terga IX + X membranous. Intermediate appendage slightly curved ventrad, short, length about one-half length of inferior appendage, basal region simple, not expanded; in dorsal view narrowly spindle-like. Mesolateral process of preanal appendage very short, apex truncate, at base broadly joined to dorsal 1/2 of mesoventral process; mesoventral process directed caudad, broadly truncate, slightly shorter than length of mesolateral process. Inferior appendage in lateral view long, slender; dorsolateral flange low, protruding as rounded triangle, without caudomesal spine; mesoventral spine present, narrow, in lateral view acute, positioned well basad; in ventral view inferior appendage narrowly triangular; mesoventral spine hidden. Phallobase very short; in lateral view apicoventral projection very broad, much shorter than diameter of apical diameter of phallobase apex, with 2 points; endothecal sclerotic band broad, ending in 2-prong hook; endothecal spines absent; phallotremal sclerite wide in dorsal aspect. Subphallic sclerite Y-shaped, arms long, pedicel short, broad; narrow in lateral view, ventrally somewhat broadened.

#### Holotype male

**: BRAZIL: Minas Gerais:** Serra do Cipó, caminho da usina, 7.vii.1974, Froehlich, Shimizu, Carvalho (UMSP000131243) (in alcohol) (MZUSP).

#### Paratypes:

**BRAZIL: Minas Gerais:** same data as holotype, except, km 110, 24.iv.1975, Froehlich, Carvalho, Shimizu, 1 male (in alcohol) (NMNH); Serra do Cipó, caminho da usina, Rio Capivara, 22.ix.1976, CGF, MAJC, GYS, 1 male, 1 female (in alcohol) (NMNH); same, except 18.xii.1973, Froehlich, 2 males (in alcohol) (NMNH); same, except 18.xii.1974, Froehlich, 1 male (in alcohol) (NMNH); same, except 18.iv.1975, Froehlich, 2 males, 4 females, (in alcohol) (NMNH); same, except afluente Rio Capivara, 19.iv.1975, Froehlich, Carvalho, Shimizu, 6 males, 2 females (in alcohol) (NMNH); Serra do Cipó, Cardeal Mota, Cachoeira Veu da Noiva, 19°18.912'S, 43°36.260'W, 800 m, 12.xi.2001, Holzenthal, Amarante, Blahnik, Paprocki, 8 males (UMSP), 1 male (in alcohol) (UMSP); Serra do Cipó, trib. to Rio Capivara, 19°14.396'S, 43°34.939'W, 1000 m, 18.ii.1998, Holzenthal & Paprocki, 1 male (MZUSP); Serra do Cipó, Mãe d’Agua, Chapeu do Sol, 19°18.807'S, 43°35.665'W, 1000 m, 17.ii.1998, Holzenthal & Paprocki, 1 male, 1 female (UFBA), 1 male, 1 female (UFRJ); km 110, Chapeu do Sol, á luz, 21.xii.1974, 1 male (NMNH); **São Paulo:** Altinópolis, Fazenda São João da Mata, Rio Baguassu, 21°00.588'S, 47°28.900'W, 745 m, 19–21.xi.2003, Holzenthal, Paprocki, Calor, 1 male (MZUSP).

#### Etymology.

Named for Serra do Cipó, the mountain range where the species was collected.

### Polycentropus verruculus

Hamilton & Holzenthal sp. n.

urn:lsid:zoobank.org:act:E3AF8858-68AA-457D-BEE3-4BFFAEA60AF2

Fig. 18 

#### Description.

A member of the *jorgenseni* species complex, Polycentropus verruculus sp. n.is separated from others of this complex by the straight, blade-like intermediate appendage, the setose lateral knob on the mesolateral process of preanal appendage, and the very compact inferior appendage with its apicomesal tooth. Like Polycentropus cipoensis, the apicoventral process of the phallobase is broad and apically emarginated, but in Polycentropus verruculus sp. n. the process is much longer and the emargination v-shaped.

##### Adult.

Length of forewing (male) 5.5 mm. Body pale brown to yellow; dorsum of head and thorax brown, clothed with long, erect brown setae; base of forewing with long, erect black setae, general vestiture of forewing with fine brown setae and many patches of pale setae scattered over surface; legs stramineous.

##### Male.

**Figure 18.**
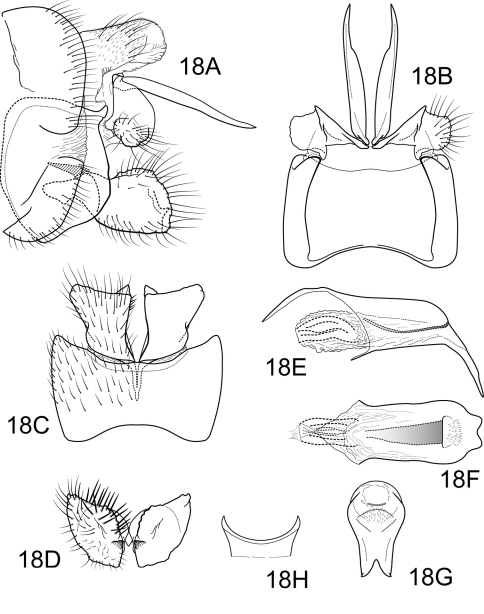
Polycentropus verruculus sp. n. Male genitalia: **A** lateral **B** dorsal **C** ventral **D** inferior appendages, caudal **E** phallus, lateral **F** phallus, dorsal **G** phallobase, caudal **H** subphallic sclerite, caudal.

Genitalia as in Fig. 18. Sternum IX in lateral view trapezoidal, slightly greater than 1/2 height of segment VIII; in ventral view slightly trapezoidal, anterior corners very sharply rounded, sides slightly convex, anterior margin moderately concave, posterior margin slightly concave with small, shallow convex medial region. Terga IX + X mostly membranous with light dorsal sclerotization basally; with numerous scattered minute spicules and several long, slender setae. Intermediate appendage straight, moderate elongate, length equal to height of sternum IX, basal region simple, not expanded; in dorsal view with basal 2/3 blade-like, apical 1/3 narrowed abruptly, apex acute. Mesolateral process of preanal appendage short, apex rounded, laterally with laterally-directed, setose, bulbous lobe. Mesoventral process of preanal appendage absent. Inferior appendage in lateral view short, quadrate; dorsolateral flange low, slightly rounded dorsally, with broad caudomesal spine, hidden in lateral view; mesoventral spine absent; in ventral view inferior appendage rhomboidal, broadest apically, caudomesal spine partially hidden, obtusely pointed. Phallobase moderately elongate; in lateral view apicoventral projection very broad, slightly longer than apical diameter of phallobase apex, with 2 points; separated by mesal v-shaped notch over apical 1/3; endothecal sclerotic band very broad, becoming less sclerotized apically; endothecal spines absent; phallotremal sclerite narrow in dorsal aspect. Subphallic sclerite U-shaped, arms short, pedicel absent; narrow in lateral view, ventrally somewhat broadened.

#### Holotype male:

**BRAZIL: Minas Gerais:** Rio Guanhães, downstream from Salto Grande dam, 19°06.289'S, 42°42.635'W, 20.x.1998, Paprocki (UMSP000046608) (MZUSP).

#### Paratype:

**BRAZIL: São Paulo:** Altinópolis, Fazenda Sáo João da Mata, Rio Baguassu, 21°00.588'S, 47°28.900'W, 745 m, 19–21.xi.2003, Holzenthal, Paprocki, Calor, 1 male (UMSP)

#### Etymology.

Diminutive form of the Latin word for wart, in reference to the wart-like structure on the preanal appendage.

### Polycentropus acinaciformis

Hamilton & Holzenthal sp. n.

urn:lsid:zoobank.org:act:17FF73EC-0951-40D8-AF4C-3094BAE1B8A8

Fig. 19 

#### Description.

Polycentropus acinaciformis sp. n.appears to belong to the *jorgenseni* species complex based on the presence of the apicoventral process of the phallobase and the endothecal sclerotic band, but it lacks the intermediate appendages and there is no evidence of the subphallic sclerite. The decurved, blade-like mesolateral process of the preanal appendage as well as the shape of the inferior appendage and the claw-like process in the endotheca distinguish this species from other Neotropical Polycentropus.

##### Adult.

Length of forewing (male) 6.5 mm. Body brown; dorsum of head and thorax brown, clothed with long, erect dark setae; base of forewing with long, erect dark setae, general vestiture of forewing with fine brown setae (in alcohol); legs brown.

##### Male.

**Figure 19.**
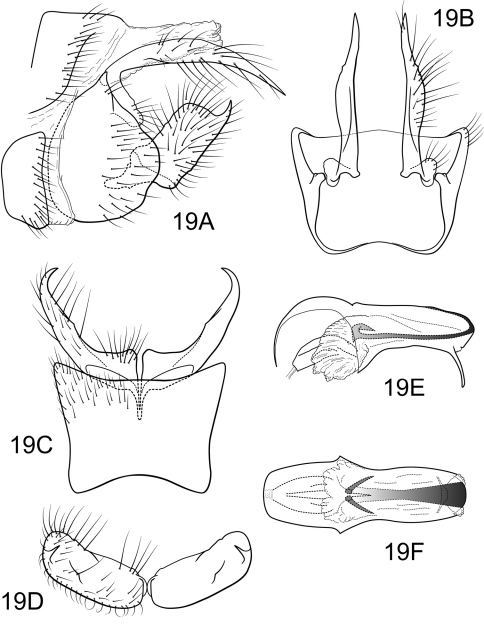
Polycentropus acinaciformis sp. n. Male genitalia: **A** lateral **B** dorsal **C** ventral **D** inferior appendages, caudal **E** phallus, lateral **F** phallus, dorsal.

Genitalia as in Fig. 19. Sternum IX in lateral view broadly subtriangular to oval, about 2/3 height of segment VIII; in ventral view slightly trapezoidal, anterior corners very sharply rounded, sides slightly constricted mesally, anterior margin moderately concave, posterior margin shallowly concave. Terga IX + X membranous. Intermediate appendage absent. Mesolateral process of preanal appendage very long, broadest at midpoint, apex acuminate, at base narrowly joined to dorsal portion of mesoventral process; mesoventral process directed ventrocaudad, digitate, less than 1/4 length of mesolateral process. Inferior appendage in lateral view short, quadrate; dorsolateral flange very low, straight dorsally, with prominent caudomesal spine, exposed in lateral view; mesoventral spine absent; in ventral view inferior appendage broad basally, slender, tapering apically, caudomesal spine prominent, acute. Phallobase moderately elongate; in lateral view apicoventral projection narrow, approximately equal to apical diameter of phallobase apex, with 1 point; endothecal sclerotic band broad, ending in 2 recurved hooks; endothecal spines absent; phallotremal sclerite wide in dorsal aspect. Subphallic sclerite absent.

#### Holotype male:

**BRAZIL: Minas Gerais:** Serra do Cipó, Capão da Mata, 19°19.347'S, 43°32.249'W, 1170 m, 13–14.ii.1998, Holzenthal & Paprocki (UMSP000033119) (MZUSP).

#### Paratype:

**BRAZIL: Minas Gerais:** Parque Estadual do Rio Preto, trib to Rio Preto, 18°06.879'S, 43°20.595'W, 700 m, 20.v.1998, Holzenthal & Paprocki, 1 male (UMSP).

#### Etymology.

From the Latin words *acinaces* (Greek *akinakes*) for scimitar and *forma* for shape, in reference to the sword-like shape of the mesolateral process of the preanal appendage.

### Polycentropus rosalysae

Hamilton & Holzenthal sp. n.

urn:lsid:zoobank.org:act:0B5B67EF-553D-4B71-B96A-A98472BB2CBC

Fig. 20 

#### Description.

Polycentropus rosalysae sp. n.is most similar to Polycentropus amphirhamphus sp. n. Both lack the intermediate appendage and have an ovoid inferior appendage with apicomesal spine, large triangular mesoventral process of the preanal appendage, and a strongly bifurcate apicomesal process of the phallobase. This species has the sclerotic endothecal band and the apicoventral process of the phallobase is broadly divided to its base. Further, the mesoventral process of the preanal appendage is broadly triangular and its mesolateral process is nearly square. These characters render it distinct from Polycentropus amphirhamphus sp. n. and other Neotropical Polycentropus.

##### Adult.

Length of forewing (male) 6.5 mm. Body brown; dorsum of head and thorax brown, clothed with long, erect brown setae; base of forewing with long, erect brown setae, general vestiture of forewing with fine brown setae and many patches of pale setae scattered over surface; legs stramineous.

##### Male.

**Figure 20.**
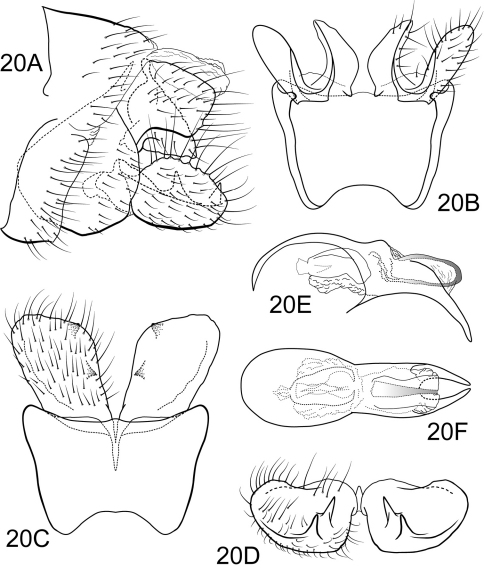
Polycentropus rosalysae sp. n. Male genitalia: **A** lateral **B** dorsal **C** ventral **D** inferior appendages, caudal **E** phallus, lateral **F** phallus, dorsal.

Genitalia as in Fig. 20. Sternum IX in lateral view broadly subtriangular to oval, about 3/4 height of segment VIII; in ventral view trapezoidal, anterior corners very sharply rounded, sides very slightly constricted mesally, anterior margin deeply concave, posterior margin slightly concave with small, shallow convex medial region. Terga IX + X membranous. Intermediate appendage absent. Mesolateral process of preanal appendage short, apex broadly truncate, at base broadly joined to mesoventral process; mesoventral process directed caudad, broadly triangular, equal in length to mesolateral process. Inferior appendage in lateral view short, generally oval; dorsolateral flange low, rounded dorsally, with prominent caudomesal spine, slightly exposed in lateral view; mesoventral spine present, narrow, in lateral view acute, positioned medially; in ventral view inferior appendage quadrate, caudomesal spine mostly hidden, acute; mesoventral spine hidden. Phallobase very short; with paired apicolateral, spur-like, decurved processes on each side of phallobase apex; endothecal sclerotic band narrow, becoming less sclerotized, broadened apically; endothecal spines absent; phallotremal sclerite wide in dorsal aspect. Subphallic sclerite absent.

#### Holotype male:

**BRAZIL: São Paulo:** Parque Estadual de Campos do Jordão, Rio Galharada, 22°41.662'S, 45°27.783'W, 1530 m, 4–5.iii.1996, Holzenthal & Guahyba (UMSP000035686) (MZUSP).

#### Etymology.

Named with great honor for the late Dr. Rosalys Guahyba, whose friendship and help were instrumental in advancing our study of the Brazilian caddisfly fauna.

### Polycentropus amphirhamphus

Hamilton & Holzenthal sp. n.

urn:lsid:zoobank.org:act:A34C4195-7E85-4C32-B6F9-D80E6FC44C5F

Fig. 21

Polycentropus  new species 8 Hamilton 1986: 149–150, 244; Fig. 7.1.

#### Description.

This new species is similar to Polycentropus rosalysae in the general shape of the preanal and inferior appendages, lack of intermediate appendage, and the bifurcate apicomesal process of phallobase. The strongly bifurcate and extreme elongation of the apicomesal process of phallobase is the most distinctive feature of Polycentropus amphirhamphus sp. n., separating if from all other Neotropical Polycentropus. Additionally, the inferior appendage lacks the mesoventral spine seen in Polycentropus rosalysae and has a narrower mesoventral process and more ovoid mesolateral process of the preanal appendage compared to that species.

##### Adult.

Length of forewing (male) 5.7–6.6 mm. Body pale brown to yellow; dorsum of head and thorax brown; with long, erect setae, area of long, pale setae on vertex of head, darker on thorax; base of forewing with long, erect dark setae, general vestiture of forewing with fine brown setae and many patches of pale setae scattered over surface; legs brown.

##### Male.

**Figure 21.**
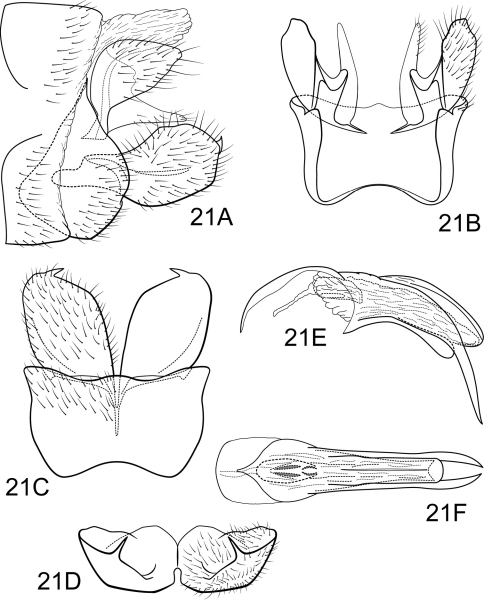
Polycentropus amphirhamphus sp. n. Male genitalia: **A** lateral **B** dorsal **C** ventral **D** inferior appendages, caudal **E** phallus, lateral **F** phallus, dorsal.

Genitalia as in Fig. 21. Sternum IX in lateral view teardrop-shaped, about 2/3 height of segment VIII; anterior corners broadly rounded, sides very slightly constricted mesally, anteriorly, anterior margin moderately concave, posterior margin slightly concave with small, shallow convex medial region. Terga IX + X membranous. Intermediate appendage absent. Mesolateral process of preanal appendage short, apex broadly triangular, at base broadly joined to dorsal 2/3 of mesoventral process; mesoventral process directed caudad, very broad basally, narrowing rapidly to slender process, slightly exceeding length of mesolateral process. Inferior appendage in lateral view short, generally round; dorsolateral flange low, rounded dorsally, with prominent caudomesal spine, partially exposed in lateral view; mesoventral spine absent; in ventral view inferior appendage approximately oval, caudomesal spine prominent, rounded. Phallobase moderately elongate; with paired apicolateral, blade-like, decurved processes on each side of slightly compressed phallobase apex; endothecal sclerotic band absent; endothecal spines absent; phallotremal sclerite narrow in dorsal aspect. Subphallic sclerite absent.

#### Holotype male:

**BRAZIL: Rio de Janeiro:** Nova Friburgo, municipal water supply, 950 m, 20.iv.1977, C.M. & O.S. Flint, Jr. (UMSP000131231) (in alcohol). (NMNH).

#### Paratypes:

**BRAZIL:** same data as holotype, 1 male (in alcohol) (NMNH); same, except 24.iv.1977, 1 male (NMNH); **Santa Catarina:** Urubici, Morro da Igreja, Cachoeira Veu da Noiva, 28°04.595'S, 49°31.090'W, 1300 m, 5.iii.1998, Holzenthal, Froehlich, Paprocki, 2 males; **São Paulo:** Parque Estadual de Campos do Jordão, Rio Galharada, 22°41.662'S, 45°27.783'W, 1530 m, 13–15.ix.2002, Blahnik, Prather, Melo, Huamantinco, 6 males (UMSP), 1 male (in alcohol) (MZUSP); Estação Biológica Boraceia, Rio Venerando, 23°39.185'S, 45°53.414'W, 850 m, Blahnik, Prather, Melo, Froehlich, Silva, 1 male (in alcohol) (UMSP).

#### Etymology.

From the Greek *amphi* for double and *rhamphos* for a curving beak or bill, in reference to the long, paired beaklike processes on the phallobase of the male genitalia.

### Polycentropus cachoeira

Hamilton & Holzenthal sp. n.

urn:lsid:zoobank.org:act:5A9E2969-A3D3-4001-BF8B-29AE7074D030

Fig. 22 

#### Description.

Polycentropus cachoeira sp. n.has some similarity to other Brazilian Polycentropus of the *jorgenseni* species complex. Most notable is the general shape of the phallus and its sclerotic endothecal band as well as the shape of the inferior appendage which resembles that of Polycentropus tripui except that it is more elongated in Polycentropus cachoeira sp. n. This species lacks the intermediate appendage and the subphallic sclerite found in other members of the species complex. Polycentropus cachoeira sp. n.is very similar to Polycentropus inusitatus sp. n. in the shape of most of the genitalic structures including a pair of large endothecal spines. The 2 species are reliably separated by details of the preanal appendage and phallus. The mesolateral and mesoventral processes of the preanal appendage are equally long in lateral view and broadly fused in Polycentropus cachoeira sp. n., while in Polycentropus inusitatus sp. n. the mesoventral process is markedly shorter and not so completely fused to the mesolateral process as seen in dorsal view. In Polycentropus cachoeira sp. n., the apicoventral process of the phallobase is undivided and there is an endothecal sclerotic band, whereas in Polycentropus inusitatus sp. n. the apex of the process is divided and the endothecal band is absent.

##### Adult.

Length of forewing (male) 5 mm. Body brown to dark brown; dorsum of head and thorax brown, clothed with long, erect brown setae; base of forewing with long, erect brown setae, general vestiture of forewing with fine brown setae and many patches of pale setae scattered over surface; legs stramineous.

##### Male.

**Figure 22.**
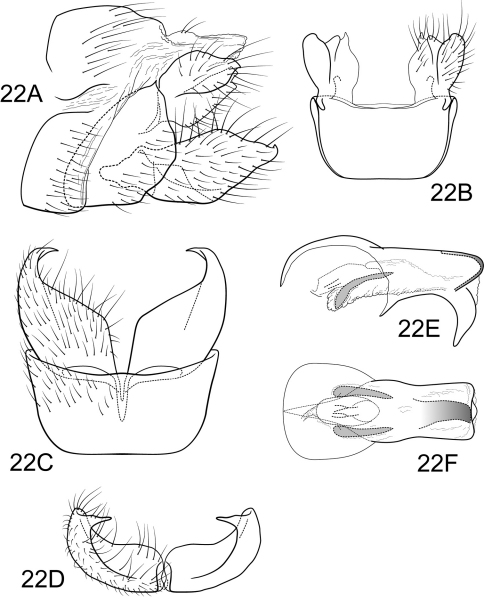
Polycentropus cachoeira sp. n. Male genitalia: **A** lateral **B** dorsal **C** ventral **D** inferior appendages, caudal **E** phallus, lateral **F** phallus, dorsal.

Genitalia as in Fig. 22. Sternum IX in lateral view broadly subtriangular, about 2/3 height of segment VIII; in ventral view slightly trapezoidal, anterior corners very broadly rounded, sides slightly constricted anteriorly, anterior margin nearly straight, posterior margin slightly concave with small, shallow convex medial region. Terga IX + X membranous, dorsum with several long, slender setae. Intermediate appendage absent. Mesolateral process of preanal appendage short, apex rounded, with acute caudal point, at base broadly joined to dorsal 1/2 of mesoventral process; mesoventral process directed caudad, size and shape of mesolateral process, equal in length to mesolateral process. Inferior appendage in lateral view moderately long, rhomboidal; dorsolateral flange low, straight dorsally, with prominent caudomesal spine, exposed in lateral view; mesoventral spine absent; in ventral view inferior appendage quadrate, caudomesal spine prominent, acute. Phallobase moderately elongate; in lateral view apicoventral projection broad, slightly longer than apical diameter of phallobase apex, with 1 point; endothecal sclerotic band very broad, becoming less sclerotized apically; with pair of large endothecal spines; phallotremal sclerite wide in dorsal aspect. Subphallic sclerite absent.

#### Holotype male:

**BRAZIL: Santa Catarina:** Urubici, Cachoeira Avencal, 28°02.839'S, 49°36.997'W, 1260 m, 6.iii.1998, Holzenthal, Froehlich, Paprocki (UMSP000033106) (MZUSP).

#### Paratypes:

same data as holotype, 2 males (UMSP).

#### Etymology.

*Cachoeira* is Portuguese for cascade or waterfall, in reference to the habitat where the species was collected.

### Polycentropus inusitatus

Hamilton & Holzenthal sp. n.

urn:lsid:zoobank.org:act:C77C7962-C590-416E-8017-3ADA378D8011

Fig. 23

Polycentropus  new species 9 Hamilton 1986: 140–142, 239; Fig. 6.45.

#### Description.

Polycentropus inusitatus sp. n.is most similar to Polycentropus cachoeira, but may be differentiated from it by the absence of the endothecal sclerotic band, the divided apicoventral process of the phallobase, and the shorter mesoventral process of the preanal appendage.

##### Adult.

Length of forewing (male) 5.5–5.8 mm. Body brown; dorsum of head and thorax dark brown; forewings nearly denuded, membrane pale brown with white areas at *r-m*, *m*, *m-cu* crossveins (in alcohol); legs paler apically.

##### Male.

**Figure 23.**
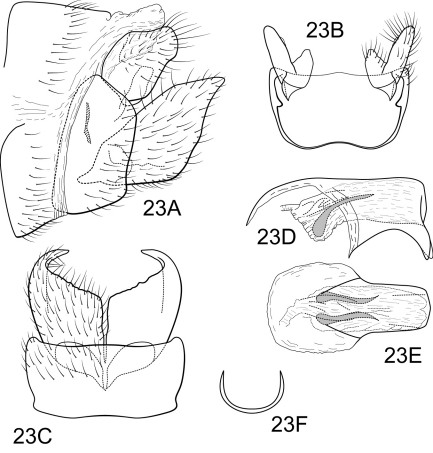
Polycentropus inusitatus sp. n. Male genitalia: **A** lateral **B** dorsal **C** ventral **D** phallus, lateral **E** phallus, dorsal **F** subphallic sclerite, caudal.

Genitalia as in Fig. 23. Sternum IX in lateral view trapezoidal, about 2/3 height of segment VIII; in ventral view slightly trapezoidal, anterior corners nearly angular, sides constriction anteriorly, anterior margin nearly straight, posterior margin slightly concave with very broad, shallow convex medial region. Terga IX + X membranous. Intermediate appendage absent. Mesolateral process of preanal appendage short, apex rounded, shorter ventrally, at base broadly joined to mesoventral process; mesoventral process directed caudad, broadly truncate, about 1/2 length of mesolateral process. Inferior appendage in lateral view moderately long, rhomboidal; dorsolateral flange low, straight dorsally, with prominent caudomesal spine, exposed in lateral view; mesoventral spine absent; in ventral view inferior appendage rhomboidal, caudomesal spine prominent, acute. Phallobase short; in lateral view apicoventral projection broad, slightly shorter than diameter of apical diameter of phallobase apex, with 2 points; separated by narrow mesal split dividing apical 1/2; endothecal sclerotic band absent; with pair of large endothecal spines; phallotremal sclerite narrow in dorsal aspect. Subphallic sclerite U-shaped, arms long, pedicel absent; very narrow in lateral view.

#### Holotype male:

**BRAZIL: Rio de Janerio:** Brejo da Lapa, Itatiaia, Coll. Museum Nacional, R. J., no date (NMNH).

#### Paratype:

same data as holotype, 1 male (NMNH).

The holotype and the paratype were sent to the senior author by Luiz S. W. Terra, Estacao Aquicola, Vila do Conde, Portugal.

#### Etymology.

Latin for rare, uncommon, or unusual, in reference to our knowledge of only 2 specimens.

### Polycentropus paprockii

Hamilton & Holzenthal sp. n.

urn:lsid:zoobank.org:act:E26A63F6-3CC4-4913-96CC-8956F7E65F03

Fig. 24 

#### Description.

Polycentropus paprockii sp. n. has several characteristics that suggest similarity to the 7 species of the *urubici* cluster. In particular, this similarity is suggested by the shape of the inferior appendage in all aspects, the notched apex of the apicoventral process of the phallobase, and the shape of the preanal appendage in dorsal view. Polycentropus paprockii sp. n.lacks the endothecal sclerotic band, but has 2 prominent endothecal spines, which are not found in these other species. The species also lacks the intermediate appendage and the mesolateral process of the preanal appendage is not digitate as in species of the *urubici* cluster.

##### Adult.

Length of forewing (male) 5–5.5 mm. Body brown; dorsum of head and thorax brown, clothed with long, erect brown setae; base of forewing with long, erect brown setae, general vestiture of forewing with fine brown setae and many patches of pale setae scattered over surface; legs stramineous.

##### Male.

**Figure 24.**
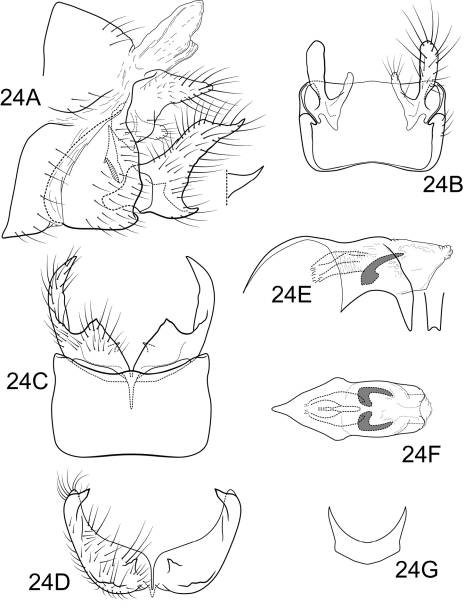
Polycentropus paprockii sp. n. Male genitalia: **A** lateral (inset, variant, inferior appendage, apex) **B** dorsal **C** ventral **D** inferior appendages, caudal **E** phallus, lateral (inset, apicoventral projection of phallobase, caudal) **F** phallus, dorsal **G** subphallic sclerite, caudal.

Genitalia as in Fig. 24. Sternum IX in lateral view subtriangular, about 2/3 height of segment VIII; in ventral view quadrate, anterior corners broadly rounded, sides slightly constricted posteriorly, anterior margin very shallowly concave, posterior margin moderately concave with small, shallow convex medial region. Terga IX + X membranous. Intermediate appendage absent. Mesolateral process of preanal appendage moderately long, apex triangular, expanded basally, at base broadly joined to dorsal 2/3 of mesoventral process; mesoventral process directed caudad, broadly digitate, about 1/2 length of mesolateral process. Inferior appendage in lateral view moderately long, somewhat triangular; posteroventral margin acute below shallow caudal emargination; dorsolateral flange low, slightly excavated medially, apically tapered to sharp, inturned point, with broad caudomesal spine, exposed in lateral view; mesoventral spine present, broad, in lateral view obtuse, positioned medially; caudomesal spine forming broad triangular base, obtusely pointed; mesoventral spine with apex visible; apex acute. Phallobase very short; in lateral view apicoventral projection moderately broad, slightly shorter than diameter of apical diameter of phallobase apex, with 2 points; separated by shallow median groove; endothecal sclerotic band absent; with pair of large endothecal spines; phallotremal sclerite wide in dorsal aspect. Subphallic sclerite U-shaped, arms long, pedicel short, broad; very narrow in lateral view.

#### Holotype male:

**BRAZIL: Minas Gerais:** Parque Estadual do Rio Preto, Rio Preto, 18°06.993'S, 43°20.373'W, 650 m, 19.v.1998, Holzenthal & Paprocki (UMSP000033123) (MZUSP).

#### Paratypes:

**BRAZIL: Minas Gerais:** Same data as holotype, 1 male (UMSP); Serra do Cipó, Capão da Mata, 19°19.347'S, 43°32.249'W, 1170 m, 13–14.ii.1998, Holzenthal & Paprocki, 1 male (UFBA); same, except 18°07’50"S, 43°20’15"W, 791 m, 12.x.2000 m, Paprocki, Amarante, Salgado, 1 male, 2 females (in alcohol) (MZUSP); same, except trib. to Rio Preto, 18°06.879'S, 43°20.595'W, 700 m, 14.xi.2001, Holzenthal & Paprocki, 1 male, 1 female (UMSP); spring trib. to Rio Macauba, near Pandeiros, 15°28.637'S, 44°44.627'W, 525 m, Paprocki & Blahnik, 1 male (in alcohol) (UFRJ); Rio São Francisco @ BR 135, 8 km S Januaria, 15°35.823'S, 44°23.396'W, 480 m, Holzenthal, Blahnik, Paprocki, Amarante, 2 males (in alcohol) (UMSP).

#### Etymology.

Named in honor of the collector, Dr. Henrique Paprocki, professor of biology at the Pontifícia Universidade Católica de Minas Gerais, Belo Horizonte, Brazil; in recognition of his contribution to our knowledge of Brazilian caddisflies.

## Supplementary Material

XML Treatment for Polycentropus boraceia

XML Treatment for Polycentropus galharada

XML Treatment for Polycentropus froehlichi

XML Treatment for Polycentropus ancistrus

XML Treatment for Polycentropus graciosa

XML Treatment for Polycentropus carioca

XML Treatment for Polycentropus fluminensis

XML Treatment for Polycentropus tripui

XML Treatment for Polycentropus soniae

XML Treatment for Polycentropus cheliceratus

XML Treatment for Polycentropus minero

XML Treatment for Polycentropus carolae

XML Treatment for Polycentropus caaete

XML Treatment for Polycentropus itatiaia

XML Treatment for Polycentropus santateresae

XML Treatment for Polycentropus virginiae

XML Treatment for Polycentropus cipoensis

XML Treatment for Polycentropus verruculus

XML Treatment for Polycentropus acinaciformis

XML Treatment for Polycentropus rosalysae

XML Treatment for Polycentropus amphirhamphus

XML Treatment for Polycentropus cachoeira

XML Treatment for Polycentropus inusitatus

XML Treatment for Polycentropus paprockii
